# Divergent patterns of endogenous small RNA populations from seed and vegetative tissues of *Glycine max*

**DOI:** 10.1186/1471-2229-12-177

**Published:** 2012-10-02

**Authors:** Gracia Zabala, Edhilvia Campos, Kranthi K Varala, Sean Bloomfield, Sarah I Jones, Hlaing Win, Jigyasa H Tuteja, Bernarda Calla, Steven J Clough, Matthew Hudson, Lila O Vodkin

**Affiliations:** 1 Department of Crop Sciences, University of Illinois, Urbana, Illinois, USA

## Abstract

**Background:**

Small non-coding RNAs (smRNAs) are known to have major roles in gene regulation in eukaryotes. In plants, knowledge of the biogenesis and mechanisms of action of smRNA classes including microRNAs (miRNAs), short interfering RNAs (siRNAs), and trans-acting siRNAs (tasiRNAs) has been gained mostly through studies with *Arabidopsis*. In recent years, high throughput sequencing of smRNA populations has enabled extension of knowledge from model systems to plants with larger, more complex genomes. Soybean (*Glycine max*) now has many genomics resources available including a complete genome sequence and predicted gene models. Relatively little is known, however, about the full complement of its endogenous smRNAs populations and the silenced genes.

**Results:**

Using Illumina sequencing and computational analysis, we characterized eight smRNA populations from multiple tissues and organs of soybean including developing seed and vegetative tissues. A total of 41 million raw sequence reads collapsed into 135,055 unique reads were mapped to the soybean genome and its predicted cDNA gene models. Bioinformatic analyses were used to distinguish miRNAs and siRNAs and to determine their genomic origins and potential target genes. In addition, we identified two soybean *TAS3* gene homologs, the miRNAs that putatively guide cleavage of their transcripts, and the derived tasiRNAs that could target soybean genes annotated as auxin response factors. Tissue-differential expression based on the flux of normalized miRNA and siRNA abundances in the eight smRNA libraries was evident, some of which was confirmed by smRNA blotting. Our global view of these smRNA populations also revealed that the size classes of smRNAs varied amongst different tissues, with the developing seed and seed coat having greater numbers of unique smRNAs of the 24-nt class compared to the vegetative tissues of germinating seedlings. The 24-nt class is known to be derived from repetitive elements including transposons. Detailed analysis of the size classes associated with ribosomal RNAs and transposable element families showed greater diversity of smRNAs in the 22- and 24-nt size classes.

**Conclusions:**

The flux of endogenous smRNAs within multiple stages and tissues of seed development was contrasted with vegetative tissues of soybean, one of the dominant sources of protein and oil in world markets. The smRNAs varied in size class, complexity of origins, and possible targets. Sequencing revealed tissue-preferential expression for certain smRNAs and expression differences among closely related miRNA family members.

## Background

High-throughput sequencing technology has become the most powerful method for global analysis of small RNA (smRNA) populations, revealing the levels of complexity in plants and other eukaryotes [1-5]. The evidence has been well documented for several endogenous smRNAs classes spawned through multiple biogenetic pathways requiring diverse molecular complexes and networks involving DICER-LIKE (DCL), ARGONAUTE (AGO), and RNA-dependent RNA Polymerase (RDR) conserved protein families [6]. In the model plant *Arabidopsis,* whose genome encodes four DCLs, ten AGOs and six RDRs, at least four endogenous smRNA-generating pathways have been uncovered so far (reviewed by [7-9]): microRNAs (miRNAs), short interfering RNAs (siRNAs), transacting siRNAs (tasiRNAs), and natural antisense siRNA (natsiRNA).

The microRNAs (miRNAs) are derived from miRNA genes consisting of inverted repeated pseudo-gene sequences that upon transcription generate double stranded RNA (dsRNA) substrates that are cut into 21-nucleotide (nt) fragments by the DCL1/AGO1 complexes. In most instances *MIRNA* genes generate multiple copies of a single miRNA that direct post transcriptional regulation of transcripts from a variety of genes. While miRNAs are evolutionary ancient, more recent duplications and inverted regions in the genome can be the source of dsRNA that also triggers the production of 21- and 22-nt short interfering (siRNAs) which also operate primarily at the post-transcriptional level.

The 24-nt class of small RNAs are also processed from dsRNA synthesized by the plant-specific RNA polymerase IVa (Pol IVa) followed by RNA-dependent RNA polymerase 2 (RDR2) reverse transcription and cutting by DCL3/AGO4(AGO6) into 24-nt siRNAs. These siRNAs arise from highly repetitive sequences, transposons, and retroelements and can also silence genes at the transcriptional level by directing DNA methylation in conjunction with AGO4 and methyl transferase HEN1 [10-12]. Unlike miRNAs, the siRNA loci produce multiple, overlapping clusters of small RNAs [6,13] generally on both strands.

The transacting siRNAs (tasiRNAs) are the products of an miRNA guided DLC4/AGO1(AGO7)/RDR6 complex that converts *TAS* transcripts from specific *TAS* genes into dsRNAs that are cut into sequential phased 21-nt fragments. The tasiRNAs target mRNAs in *trans* in a fashion resembling miRNAs [14-16]. Finally, the natural-antisense siRNA (nat-siRNA) are 21-nt fragments resulting from the DCL2/AGO/RDR6 action on dsRNA sense- and antisense-transcript pairs induced by abiotic or biotic stress. They have been shown to be post-transcriptional regulators of genes involved in pathogen defense and stress responses in plants [17,18].

The high throughput sequence outcomes from different plant species have revealed the great abundance and complexity of these smRNA endogenous populations. The focus has been those sequences with high repetitions ranging in size between 21 to 24 nt. In turn, the smRNA sequence length has become an indicator of the DCL enzyme and the pathway generating them. The miRNAs are mostly 21 nt and the result of the DCL1 containing complex. On the other hand, the majority of the siRNAs representing highly repetitive elements are processed from double stranded RNA (dsRNA) synthesized by the plant-specific RNA polymerase IVa (Pol IVa) followed by RNA-dependent RNA polymerase 2 (RDR2) reverse transcription and diced by DCL3 into 24-nt siRNAs. Other siRNAs, 21 nt in length, are derived from genomic regions with inverted repeats or inverted repeated genes. These siRNAs are most likely processed from single-stranded hairpin RNA precursors in a process resembling miRNA biogenesis. Some of these siRNAs might represent “evolving miRNAs” that target endogenous genes with sequence similarity. Recently, it was discovered that low abundance 22-nt miRNA/transacting siRNA products of DCL1/DCL2) bind to specific AGO/DCL/RDR2 complexes to produce dsRNAs and a second wave of phased secondary (or transitive) siRNAs from the regions surrounding their target sites [19]. The 21-nt tasiRNAs are the product of miRNA-directed fracturing of *TAS* transcripts via the pathway involving the DCL4/RDR6 complex [16].

Three of the six *Arabidopsis* RDRs (RDR1, RDR2 and RDR6) have orthologs in many plant species, an indication that these proteins are conserved [20]. The functions of RDRs depend on the DCL (1-4)/AGO (1-10) complex they recruit. One would expect that the larger and more complex a plant genome is, the higher the diversity in effector proteins may be. As evidence, the rice plant has five DCLs and 19 AGO family members belonging to seven families [21,22]. This variability between these two plant species may suggest the likelihood that plants with even larger repetitive genomes (>60%), such as soybean and maize, may have evolved additional variation in the components of gene silencing complexes, the smRNA classes they generate, and the genes that are regulated and silenced. Initial computational estimates have identified seven DCL homologs in soybean including highly conserved homologue copies of the four canonical DCLs which may be the result of two to three rounds of genome duplications in the last 45 million years [23]. An example of the added smRNA complexity found so far in soybean is the large collection of siRNAs (21 and 22 nt) that silence the nine members of the chalcone synthase (*CHS*) gene family in soybean seed coats [24]. In maize, on the other hand, a significantly larger number of 22-nt siRNAs have been found in root and shoot tissues that function differently from the 21- and 24-nt siRNAs based on their origin and targeted genes [4]. In contrast, this 22-nt siRNA class is lacking in other monocots such wheat, barley and rice [25].

Global smRNA sequencing studies in several plant species have shown shifts in the pools of the different smRNA classes in various tissues/organs. In general, plant cells have a small number of unique and highly abundant 21-nt miRNAs and a large number of diverse siRNAs, mainly 24-nt in size [1]. In *Arabidopsis* beside finding smRNA differences in tissue expression, the siRNA population as a whole is larger in inflorescence than in seedlings suggesting that PolIVa may be developmentally regulated [9,26].

In maize where >85% of the genome consists of transposable elements [27], the largest numbers of reads were 21 nt in the root while in the shoot the most abundant reads were 24 nt. The 22-nt size class had significant number of repetitions in both tissues and based on the origin and targeting region of genes it was proposed that the 22-nt siRNAs function differently from the 21- and 24-nt siRNAs [4].

To date, four small RNA sequencing studies focused mainly on miRNAs have been reported for the soybean (*Glycine max*) [28-31]. Here we document the small RNA sequence populations obtained through the Illumina short-read SBS (sequencing by synthesis) method [32] in multiple tissues of the yellow seed soybean varieties (early development whole seed, seed coat, cotyledons, germinating cotyledons, shoot and leaf stages) and the seed coat of a black seed variety. Each tissue’s smRNA population was sorted into the different smRNA groups (miRNA, siRNA) based on annotations of existing databases, sequence length, abundance and tissue specific expression. The genomic origin of numerous miRNAs and possible targets were determined by mapping to the soybean genome and predicted cDNA gene models. A comprehensive analysis of the family members of all miRNAs present in these seed and vegetative tissues is presented. Tissue differential expression based on the flux of normalized miRNA and siRNA abundance in the eight smRNA libraries was prevalent, some of which was confirmed by small RNA blotting. We also focused on siRNAs and their associated genes as well as those derived from a largely repeated 26S ribosomal DNA (rDNA) containing genomic sequence and those matching transposon sequences. Evidence is also presented for two *G. max TAS3* genes and derived tasiRNAs. Comparison of seed and non-seed tissues revealed that the size classes of small RNAs varied amongst different tissues with the developing seed and seed coat having greater numbers of unique small RNAs of the 24-nt class compared to the vegetative tissues of germinating seedlings.

## Results

### Sequencing and computational analysis of small RNA populations from multiple soybean tissue and organ systems

Given the increasingly diverse and significant role endogenous non-coding smRNAs play in gene regulation, development, and response to biotic and abiotic stress, we determined the size and composition of smRNA pools in multiple organs of the soybean plant. The tissues/organs selected included very young (12-14 days after flowering) whole seed, and dissected seed coats and cotyledons from mid-maturation embryos, as well as the cotyledon, stem and leaf from germinating or adult tissues (Table 1 and Materials and Methods for details). The low molecular weight (LMW) or total RNA extracts were fractionated further at Illumina following their preparative protocol for SBS sequencing on the Illumina Genome Analyzer (see Materials and Methods).

**Table 1:** Summary of small RNA sequencing from diverse tissues and organs of soybean

**Tissue/Organ**^**a**^	**Stage**^**a**^	**Line**^**b**^	**Library code**	**GEO sample number**^**c**^	**Total reads (Millions)**	**Number of uniques**^**d**^	**Number matching miRBase**^**e**^	**Percent match to genome**^**f**^
Whole seed	12-14 DAF	Williams	WS	GSM899820	3.0	38749	393	84
Seed coat	Immature	Richland	SCR	GSM543393	2.9	32846	475	85
Seed coat	Immature	Williams 55	SCM	GSM543396	6.1	35413	263	92
Seed coat	Immature	Williams	SCW	GSM543394	2.9	27343	525	87
Cotyledon	Immature	Williams	Cot	GSM543395	3.0	27288	390	85
G. Cotyledon	7 day seedling	Williams	GCot	GSM899821	12.5	38961	876	80
Stem	2 week plants	PI194639	ST	GSM899822	4.2	37539	486	59
Leaf	1 month plants	PI462312	LE	GSM899823	6.1	6855^d^	273	70

^a^Whole seed at 12-14 DAF (days after flowering); seed coat and cotyledon samples are dissected from immature, mid-maturation, green seed at fresh weight range of 50-75 mg per seed; G, germinating cotyledon from 7-day etiolated seedling; Stem, of 2-week-old plantlets, Leaf, first or second fully expanded trifoliates from 1-month-old plants.

^b^The standard Williams variety has the *i*^*i*^ genotype that specifies pigment present in the hilum with an otherwise yellow, non-pigmented seed coat. Williams 55 is a pigmented seed coat mutant line in Williams that contains the homozygous recessive genotype (*i/i*) at the *I* locus and Richland has the dominant, fully yellow *I/I* genotype.

^c^Sample accession numbers for sequence samples entered into Gene Expression Ominbus (GEO) (http://www.ncbi.nlm.nih.gov/geo/). Accession numbers to be determined.

^d^The number of unique signatures after adapter trimming are adjusted to account for increased sequencing output. The unique signatures with less than 5 reads were eliminated from libraries with approximately 3 million reads; similarly uniques with less than 10 counts were eliminated in the seed coat (Williams 55) and leaf libraries and less than 16 reads were eliminated from the germinating cotyledon library. As will be discussed in the results, the leaf library (LE) was found to contain a single superabundant miRNA signature that composed 65% of the 6.1 million total reads, thus reducing the overall diversity of unique signatures.

^e^miRBase (http://microrna.sanger.ac.uk/) the curated mature miRNA database maintained at the Sanger Institute.

^f^Percent of unique matching the Glycine max genome at Phytozome [33].

As summarized in Table 1, a total of nearly 41 million raw reads were obtained from the eight libraries. The number of unique reads from all libraries amounted to a total of 244,994. This number was collapsed to 135,055 unireads after subtracting overlapping sequences among the eight libraries under study. Each smRNA population consisted of between 6,855 - 38,961 unique reads (Table 1). These figures may represent the overall extent of the soybean small RNA population complexity for the soybean tissues studied at the specific developmental stages chosen and under optimal environmental conditions. Additional file 1 presents all 135,055 unireads, their annotations, and the normalized read counts in each library. The majority (107,278) or an average of 80% of the unique smRNA signatures mapped to the current 8X soybean genome sequence (Williams 82, an isoline of Williams) at the Phytozome database [33] using Novalign [34] as shown in Additional file 1 while relatively few (1,804) matched to the Sanger miRNA database (miRBase) [35]. Many sequences were not represented in either miRBase or the nt (nucleotide)-NCBI database [36].

### Size distribution of the smRNA populations reveals differences in seed and vegetative tissues

**Figure 1:**
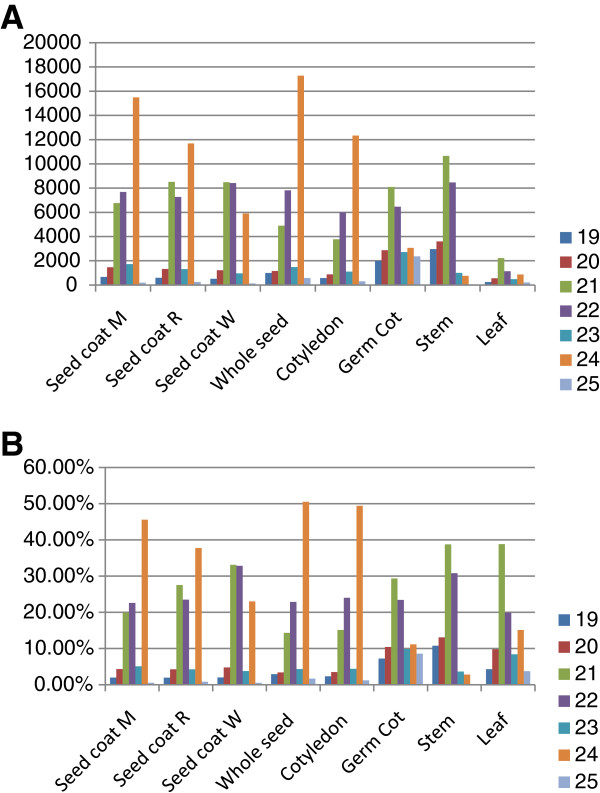
**Size distributions of smRNA sequence signatures in each tissue/organ library of soybean.** The unique signatures having lengths of 19-25 nucleotides were graphed as shown in the color code. (**A**) Sizes of the actual number of unique signatures. (**B**) Percentage of the total unique signatures. See Table 1 for library descriptions.

The sequence length of a small RNA is a predictor of its biogenetic pathway and functionality in accordance with its corresponding smRNA class and thus, examining the range of sizes of the unireads in each population sample is informative. The smRNA signatures ranging in length between 19 and 25-nt were taken into account for the schematic size distribution in each tissue sample population shown in Figure 1A and B. The 24-nt smRNAs were the most prominent class in the seed (immature coat and cotyledon) libraries while in the organs from germinating or adult plants (stem, leaf and germinating cotyledon) the 21-nt smRNAs had the largest number of unique reads (Figure 1A and B). Second in abundance in the seed smRNA libraries were the 21-nt smRNAs followed very narrowly by the 22-nt smRNAs. Interestingly, in the very young whole seed and cotyledon smRNA libraries the 22-nt signatures were more abundant than the 21-nt signatures. The significance of these results is addressed in the discussion section.

### Differential expression of miRNAs and identification of putative targets

**Figure 2:**
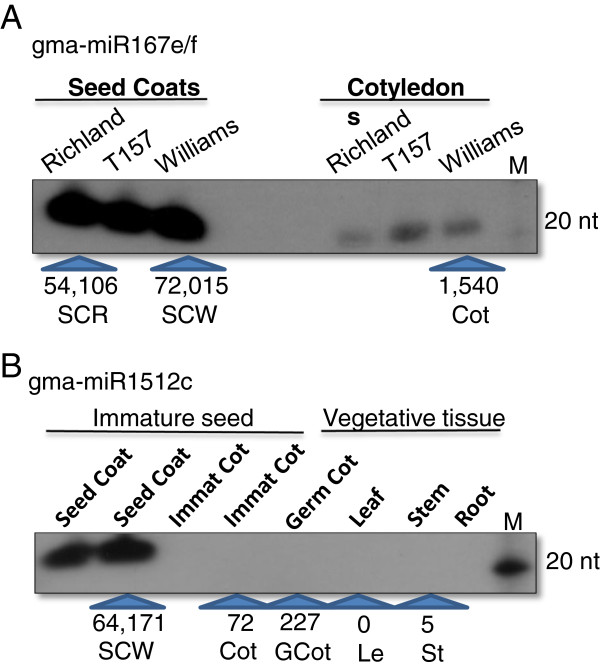
**Differential tissue accumulation for two miRNAs.** (**A**) SmRNAs hybridizing to an oligoprobe representing the soybean miR167 family (Table 2, #1) manifested higher expression in the seed coats in all cultivars examined. T157 is a mutant isoline of Richland with pigmented seed coats. (**B**) SmRNAs hybridizing to an oligoprobe representing the gma-miR1512c (Table 2, #4) were expressed almost exclusively in the seed coats of immature versus vegetative tissues of Williams. The number of sequence counts per million as shown in Table 2 in each smRNA library is given at the bottom of the two panels for comparison of the blot results to the sequencing results. Note: the blot results for the stems and leaves are from Williams whereas the sequencing results are from PI lines shown in Table 2. M designates radiolabeled Decade™ Markers with the 20 nucleotide size shown.

Closer scrutiny of the number of occurrences of signatures best representing each miRNA in the different smRNA tissue libraries revealed differential expression patterns for some that were selected for further study. Table 2 presents the normalized number of reads (counts) per million total reads for the expression of 23 soybean miRNAs in the eight smRNA libraries. It is clear that there is wide disparity in the number of reads representing individual family members. More interestingly, several of the miRNAs were abundant in either the immature seed coats or cotyledons and not in the vegetative tissues such as gma-miR167e/f (#1) and gma-miR1512c (#4). The highly preferential expression in immature seed coats was confirmed for both gma-miR167e/f (#1) and the gma-miR1512c miRNA (#4) by small RNA blots (Figure 2).

**Table 2:** Differential expression of soybean miRNAs

				**Normalized counts in each library per million reads**^a^										
	**Sequence**	**L**	**miRBase**	**Immature seed tissue**					**Vegetative tissue**			**miRNA gene origins**^**b**^	**Target glyma cDNAs**	**Annotation**
				**WS**	**SCR**	**SCM**	**SCW**	**Cot**	**GCot**	**ST**	**LE**			
1	TGAAGCTGCCAGCATGATCTT (see Figure 2A blot)	21	gma-miR167e/f	**48472**	**54106**	**9009**	**72015**	1540	**8512**	5	224	Gm20:37,901,914	20g02540.1	None
												Gm10:46,574,320	10g35160.1	Unknown function
2	TGAAGCTGCCAGCATGATCTG	21	gma-miR167c	65	412	37	281	782	**9252**	0	673	Glyma10g30370.1	20g02540.1	None
												Glyma20g36720.1*		
3	TGAAGCTGCCAGCATGATCTA	21	gma-miR167a/b	225	52	14	76	597	400	15	18	Glyma19g34240.1*	18g05330.1	Auxin response factor
												Gm10:2,640,290	02g40650.1	“
												Glyma02g16230.1*	11g31940.1	**“**
												Glyma03g31410.1*	14g38940.1	**“**
4	TAACTGAACATTCTTAGAGCAT (see Figure 2B blot)	22	gma-miR1512c	**94395**	**36802**	**44554**	**64171**	72	227	5	0	Gm02:8609718	08g17790.1	Cysteine-rich receptor- like kinase
													10g21350.1	Villin
5	CAGCCAAGGATGACTTGCCGG (see Figure 3A blot)	21	gma-miR169a	14	8	12	18	0	2042	7	32	Gm10:40,332,893	14g01360.1	Nuclear transcription factor
												Glyma09g28810.1*		
6	TGACAGAAGAGAGTGAGCAC**_**	20	gma-miR156a	3331	2150	**8462**	2190	**20788**	**22007**	**38342**	**31850**	Gm17:4,291,733	05g38180.5	Squamosa PB-like TF
												Glyma17g08280.1*	08g01450.1	“
												Glyma17g33830.1*	06g17700.1	“
												Gm14:9,431,608	04g37390.1	“
												Gm13:20,521,472	05g00200.1	“
												Gm06:4,013,580	17g08840.1	“
												Gm04:4,257,067	03g27200.1	“
												Gm02:41,864,174	19g32800.1	“
7	TTGACAGAAGA**T**AGAGAGCAC	21	gma-miR156c/d/e/g	290	4381	2889	2053	25	**14252**	**13237**	**5014**	Gm19:8,895,400	19g26390.1	Squamosa PB-like TF
												Gm09:37,843,837	13g24590.1	“
												Gm08g05430.1*	07g31880.1	“
												Gm07:9,347,149	18g36960.1	“
												Gm02:7,812,536	02g30670.1	“
												Gm08:3,891,372	19g32800.1	“
												Gm16:5331711	03g29900.1	“
8	TTGACAGAAGA**G**AGAGAGCAC	21	gma-miR156b/f	214	1631	3171	2018	**98674**	**42110**	9	51	Gm14:990,353	11g36980.1	Squamosa PB-like TF
	(see Figure 3A blot)											Gm02:50,779,324	18g36960.1	“
												Gm06:19,264,024	02g30670.1	“
													19g32800.1	“
													03g29900.1	“
9	TCGCTTGGTGCAGGTCGGGAA	21	gma-miR168b	1749	3565	1891	3244	1148	2935	1189	731	Glyma09g35160.1		
	(see Figure 3A blot)											Gm01:48,070,389		
10	TCGGACCAGGCTTCATTCCCC (see Figure 3A blot)	21	gma-miR166a-3p	4471	**11557**	**16665**	**29735**	**12361**	**5812**	**24375**	**5460**	Glyma16g02380.1*	08g21620.1	HD-zip transcription factor
												Glyma02g15860.1*	07g01940.1	“
												Glyma04g38430.1*	08g21610.1	“
												Glyma08g00630.1*		
11	GGAATGTTGTCTGGCTCGAGG	21	gma-miR166a-5p	10	154	500	252	230	45	23	182	Glyma16g02380.1*		
												Glyma08g00630.1*		
												Glyma04g38430.1*		
12	TGAGACCAAATGAGCAGCTGA (see Figure 3B blot)	21	gma-miR3522	3987	1849	4478	2524	**112139**	**43366**	1478	**646995**	Glyma15g06080.1*	07g31270.1	Tyrosinase
13	AGGGATAGGTAAAACAA**CT**AC (see Figure 3B blot)	21	gma-miR1510b-5p	891	3293	**16652**	1798	**9906**	2809	2825	2028	Glyma02g08410.1*		
14	AGGGATAGGTAAAACAATGAC	21	gma-miR1510a-5p	428	0	**5633**	699	2628	221	0	0	Glyma16g27510.1*		
15	TGTTGTTTTACCTATTCCACC	21	gma-miR1510a/b-3p	31	20	65	18	132	34	9	0	Glyma16g27510.1*	04g39740.1	Disease-resistance protein
												Glyma02g08410.1*	18g14660.1	“
													19g07700.2	"
16	TCTCATTCCATACATCGTCTGA (see Figure 3B blot)	22	gma-miR1507a	**29315**	**17502**	**5373**	**15728**	**17964**	**19959**	**34871**	**10750**	Glyma17g08340.1* Glyma13g22280.1*		
17	AGCTCTGTTGGCTACACTTT**G**	21	gma-miR394a	19	693	2382	782	15	0	2412	11	Glyma17g38110.1		
18	TTCCACAGCTTTCTTGAACTG	21	gma-miR396a/e/h/i -3p	53	144	27	120	0	73	388	52	Glyma13g22840.1*	19g39020.1	Unknown function
												Gm17:35,366,632	03g00550.1	Retrovirus related Pol
												Gm14:13,971,439	13g22840.1	polyprotein
												Gm17:9,044,867		none
19	AGAATCTTGATGATGCTGCAT	21	gma-miR172a/b	2	32	10	25	8	30	349	616	Gm20:40,895,751	12g07800.1	AP2-like transcription factor
												Glyma13g39860.1*	11g15650.1	“
												Gm12:6,110,723	15g04930.1	“
												Gm10:31,592,580	13g40470.1	“
												Gm10:43,474,799	02g09600.1	“
20	CAGGGGAACAGGCAGAGCATG	21	gma-miR408c/a-5p	5	0	11	0	22	3	12	4669	Glyma10g27760.1*	11g01200.1	DNA-directed RNA
												Gm02:837,430		polymerase
21	TTGGCATTCTGTCCACCTCC	20	gma-miR394c-5p	286	102	71	147	13	2	412	0	Glyma17g38110.1*	18g00870.1	F-box protein
												Gm15:3,767,188	05g28050.1	“
												Glyma14g39920.1*	08g11030.1	“
												Gm08:9,880,084	11g36960.1	“
												Glyma06g02270.1*		
												Glyma05g30360.1*		
22	TAGAAAGGGAAATAGCAGTTG	21	gma-miR1508a/b	464	187	52	120	913	**7892**	704	1063	Gm16:32,903,801	09g30500.1	Pentatricopeptide
												Gm09:28,530,237	16g27800.1	repeat-containing protein
													09g39260.1	“
													09g39250.1	“
23	TAAGAGAATTGTAAGTCACTG	21	gma-miR4413	32	29	33	46	72	109	73	91	Glyma19g02070.1	09g07250.1	Pentatricopeptide
													07g11410.1	repeat-containing protein
													07g11290.1	“
													16g31950.1	“
													09g07300.1	“
													09g30620.1	“

^a^Normalized read counts higher than 5000 are in bold. Library abbreviations are in Table 1. ^b^The position of the miRNA hairpin in the *Glycine max* reference genome or the associated gene Model number is shown. *Low confidence gene model. L= sequence length. Underline indicates base difference between family members.

**Figure 3:**
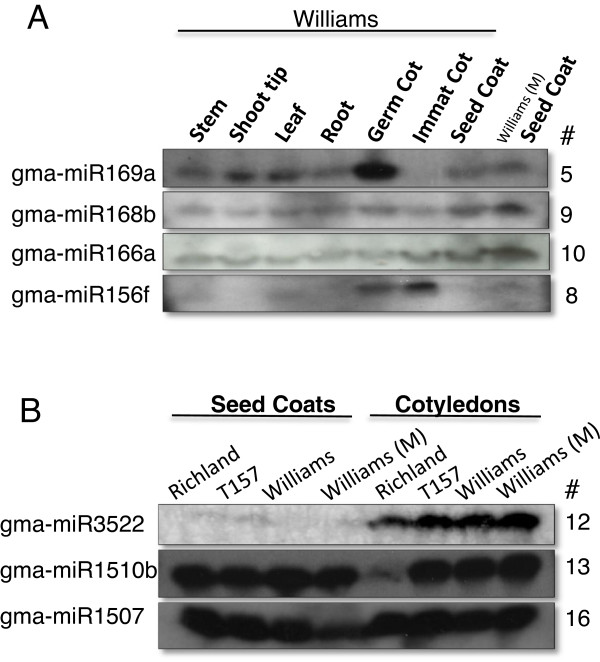
**Varied levels of tissue expression for seven soybean miRNA families.** SmRNAs were hybridized to oligoprobes representing: (**A**) miR169a (Table 2, #5), miR168b (Table 2, #9), miR166a (Table 2, #10) and miR156 (Table 2, #8) in multiple tissues of Williams. (**B**) gma-miR3522 (Table 2, #12), gma-miR1510b (Table 2, #13) and gma-miR1507a (Table 2, #16) in seed coats or cotyledons of Richland, T157 (mutant pigmented isoline of Richland) and Williams lines. Williams (M) is mutant line 55 that has pigmented seed coats.

A smRNA (#5 of Table 2) that matched gma-miR169a had its highest levels in the germinating cotyledons compared to the seed or other vegetative tissues and this was confirmed with an RNA blot shown in Figure 3A. On the other hand, gma-miRNA168b (#9) and gma-miR166a-3p (#10) were present in all the tissues examined as measured by both sequencing and blotting experiments (Table 2 and Figure 3A). Interestingly, another miRNA166 sequence, gma-miR166a-5p (#11), mapping at the 5’ end of the precursor RNA stem loop, accumulated at very low levels in all the tissues, in contrast with the high level accumulation of the gma-miR166a-3p derived from the 3’ end of the same RNA precursor (#10 in Table 2).

Table 2 shows several members of the well-known miR156 family in soybean including one (gma-miR156a) that accumulated to relatively high levels in all of the tissues (sequence #6 in Table 2). But the most surprising result was the differential tissue expression for two miRNAs of the same miR156 family with identity to gma-miR156c/d/e/g and gma-miR156b/f, whose sequences differed only in one nucleotide transition of T– > G (Table 2 sequence #7 versus #8). The gma-miR156c/d/e/g sequence (with a T nucleotide) had only 25 reads in the immature cotyledon while the gma-miR156b/f family members (G) had 98,674 reads in the same tissue. Opposite expression was observed in the stem library for the two family members (13,327 versus 9 reads). As shown in Figure 3A, a 21-mer probe reflecting the gma-miR156b/f family members (sequence #8 of Table 2) revealed a high level in the immature cotyledons as expected. A probe representing sequence #7 with the single nucleotide difference also showed the same pattern since family members that differ by only a single base pair cannot be distinguished by hybridization (data not shown). These results are clear evidence of the advantages of analyzing the abundance of specific smRNAs by number of sequence reads rather than by signal strength in gel-blot autoradiographs. The sequencing data unveiled the differential expression of the two sets of miR156 family member mapping to different locations in the soybean genome (Table 2).

Table 2 also illustrates that sequence #12, which is similar to the recently identified gma-miR3522 [37], was highly abundant in the immature cotyledon, germinating cotyledon, and leaf, but not the immature seed coats. In fact, miR3522 accounted for an astonishing 65% of the 6.1 million total reads in the leaf sample and 11% of the total reads in the immature cotyledon sample. Figure 3B confirms its differential expression between the immature cotyledons and seed coats from two different cultivars. On the other hand, the recently identified soybean families gma-miR1510 and gma-miR1507 [28-31] have specific family members (#13 and #16 in Table 2) that were expressed at moderate to high levels in most tissues. Finally, several other miRNAs (sequence #s 5, 18, 19, 20, 21, 22, 23) showed differential expression highest in vegetative tissues of either, the germinating cotyledon, stem, or leaf relative to the immature seed tissues although the numbers of sequence reads were low overall for even the family member with the highest read count.

Table 2 represents only a small selection of the miRNA family member sequences and their abundances in the different tissue libraries. The full complement of 4,031 sequences representing about 213 different miRNAs from a total of 117 families is shown in Additional file 2 which also presents the normalized number of reads found in each tissue for each sequence. This data set shows all the related miRNA families and minor sequence or nucleotide variants. Generally, the sequence(s) with more abundant reads within each family are likely to be the authentic miRNAs. The other sequences that vary in length or have nucleotide differences could be produced biologically during processing steps in the generation of the miRNAs or from sequencing errors in the high throughput format.

### Distinguishing the origins and genes targeted by miRNAs through alignment to the soybean genome and prediction of hairpin repeats

**Figure 4:**
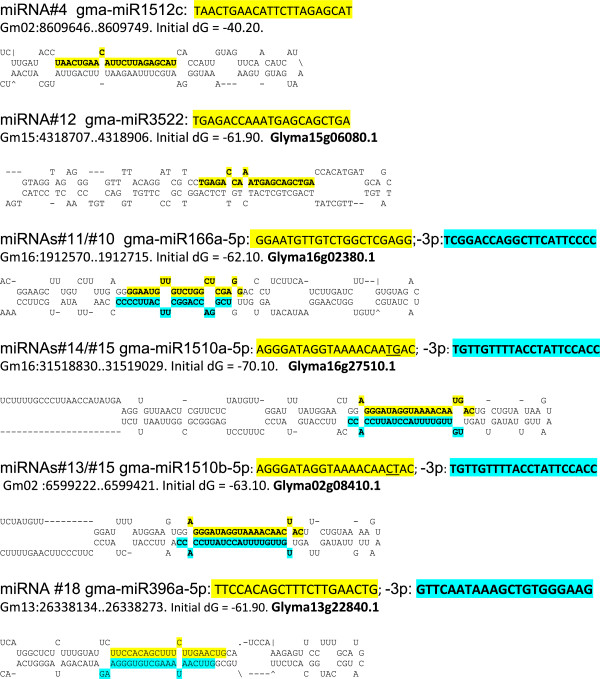
**Stem loop secondary RNA structures of differentially expressed miRNAs.** Eight differentially expressed miRNAs presented in Table 2 are mapped and highlighted on the folded precursor RNA sequence. Highlighted yellow are the 5p miRNAs (#4, #12, #11, #14, #13, #18) and in aqua, the 3p miRNAs (#10, #15 and gma-miR396a-3p). The location in the soybean genome (Gm number) and any low confidence Glyma gene model numbers (Glyma number) associated with the miRNA genes by Phytozome are shown.

The miRNA genes are constantly evolving and tend to originate from transcribed repeated sequences or pseudo-genes when transcription products fold into stable secondary structures. The majority of miRNAs are 21 nt in size, clipped from the stem portion of hairpins by DCL1/AGO1 complexes. In general, many identical copies of a single miRNA are clipped off the same strand location of a given pre-miRNA transcript. In most cases, miRNAs demonstrate a strand bias with only one side of the hairpin producing the miRNA that associates with the AGO complex and the miRNA* representing the opposite strand is unstable. However, in some instances miRNA*s of the opposite strand have been recovered and are designated with a 5p and 3p designation. In order to define the origin of the miRNA, the precursor region that is generally in the range of 200 nt must form an energetically stable hairpin loop. In Table 2, we present the putative origins of 23 miRNA genes in soybean by the genomic locations that produce stable hairpin RNA secondary structures. The gma-miR1512c expressed very highly in seed coats (sequence #4) maps to a genomic position Gm02:8609718 of the 8X soybean genome model [33] as illustrated by the hairpin structure shown in Figure 4. This site was not associated with any computationally predicted soybean gene models (known as Glyma models) from Phytozome. The only other matches for this miRNA (sequence #4) and thus possible targets are Glyma08g17790.1 which is predicted to encode a cysteine-rich receptor-like kinase and Glyma10g21350.1 having the annotation of villin.

In contrast to gma-miR1512c which is abundant in the seed coats only, the miR156 family member sequences (#6, #7, #8 in Table 2) map to at least 15 positions in the soybean genome that do not encode proteins but are able to form authentic hairpin structures and can be origins of these miRNAs. The targets of the miR156 family are numerous gene models encoding squamosa promoter-binding-like proteins (SBP) as known from other well studied systems [38]. Other miRNA targeted genes include cDNA models annotated as auxin response factor, HD-zip transcription factor, and Ap2-like transcription factor. Some of the genomic origins for the miRNAs fall into regions of the Phytozome soybean genome assembly that have been annotated as low confidence gene models (those marked with an asterisk in Table 2). Since all of these contain repeats able to form hairpins they are most likely miRNA genes instead of protein coding genes. Additional file 1 and Additional file 2 contain the complete information for the 213 miRNAs sequences, their genomic locations, and the Glyma models potentially targeted by them. Figure 4 shows the predicted hairpin structures of some of the miRNAs in Table 2. As depicted for miRNAs #10 and #11 hairpin pair, generally one of them had significantly more counts associated with it than the one at the other end and in most cases the processing of the miRNA from either side of the loop was staggered by 2-nt (Figure 4).

### Sequence alignments distinguish miRNAs and siRNAs

Of the 107,279 smRNA sequences found to match the 8X soybean genome database from Phytozome [33], the great majority had no matches in miRBase. These could potentially represent new soybean miRNAs or siRNAs that could silence soybean genes. Using the list of all unique smRNAs in the eight libraries ( Additional file 1), we first grouped these sequences based on their annotations and the groups containing the highest number of signatures were examined to ascertain their origin (miRNAs or siRNAs) and to predict their targets. Although a few analyses of soybean miRNAs have been reported recently [28-31], this is the first report on the global view of the siRNA fraction of the small RNAs found in multiple soybean tissues. The siRNAs may result from secondary amplification of transcripts targeted by miRNAs. Alternatively, siRNAs may be generated from inverted sequence repeats in the genome in a manner similar to that of the naturally occurring *CHS* (chalcone synthase) siRNAs generated by the soybean *I* locus that controls seed coat color in soybean [24]. Our previous examination of the seed coat libraries from yellow seed coats (libraries SCR and SCW), which accumulate abundant *CHS* siRNAs compared to the mutant pigmented seed coat (SCM) and the cotyledon library (Cot) that do not have *CHS* siRNAs, provided the data to demonstrate the genotype and tissue production of *CHS* siRNAs. Below we describe several examples of other siRNAs gleaned from the eight libraries that potentially could downregulate complementary genes.

#### Pentatricopeptide repeat (PPR)-containing protein

**Figure 5:**
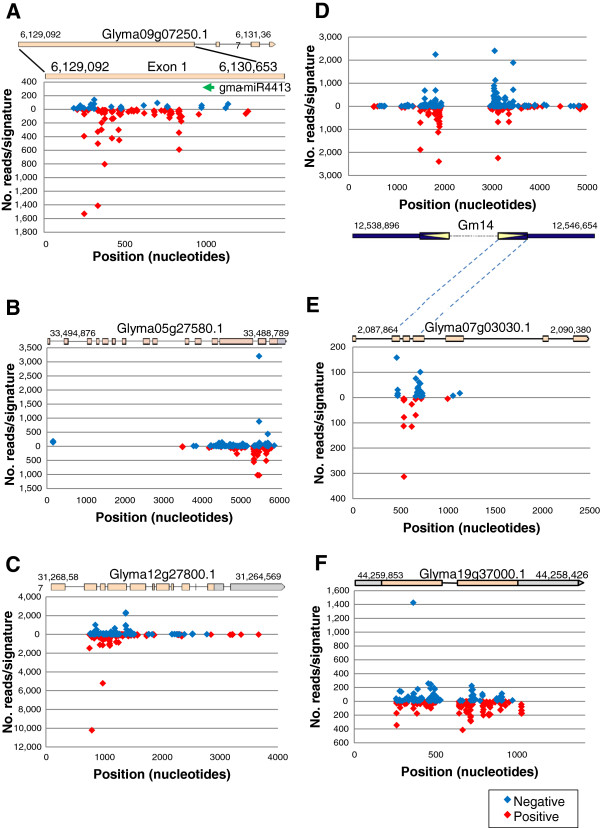
**siRNAs aligned to soybean genomic sequences.** Collections of smRNAs with the same annotation were aligned to specific genomic sequences with Bowtie. (**A**) Pentatricopeptide repeat-containing protein (PPR) siRNAs to Glyma09g07250.1. (**B**) Auxin response factor (ARF) siRNAs to Glyma05g27580.1. (**C**) TIR domain resistant gene (TIR-NBS-LRR) siRNAs to Glyma12g27800.1. (**D**) Aquaporin siRNAs aligned to an inverted repeat with similarity to an aquaporin gene NIP6-1, Glyma07g03030.1 shown in (E). (F) Aquaporin siRNAs unrelated to those in D and E aligned to another aquaporin transporter, Glyma19g37000.1. Blue diamonds represent siRNAs aligning to the negative strand and red diamonds to the positive strand. Green arrow in (A) marks the binding site of gma-miR4413. Dashed lines connect the region of similarity in Glyma07g03030.1 (**E**) to the aquaporin inverted repeat (D).

A group of signatures annotated as pentatricopeptide repeat-containing protein (PPR) had different origins, with some of the signatures originating from miRNAs and others being siRNAs. The most abundant signature (#22 in Table 2) had similarity to gma-miR1508a and gma-miR1508b and was expressed in all tissues examined with the highest level (7892 occurrences) observed in germinating cotyledons. This miRNA maps to two loci of origin [chromosome 16 and 19 (Gm16, Gm19)] and potentially targets four different Glyma cDNAs annotated as PPR repeat containing protein. Also in this annotation group was a smRNA (#23, Table 2) that matches an inverted repeat region on Gm19 capable of forming a secondary structure with dG = -61.60 and similar to gma-miR4413. It is expressed at relatively low levels in all tissues and potentially targets six different Glyma gene models having the PPR motif. Further inspection using Bowtie [39] (or *MultAlin*[40]) found a series of 115 unique siRNAs matching either the sense or antisense strand of the Glyma09g07250 PPR gene’s first exon (Figure 5A).

All together, these results revealed the existence of three miRNA genes, miR1508a, miR1508b, and miR4413 that are likely regulators of a group of genes that contain PPR motifs. PPR proteins are sequence-specific RNA-binding proteins involved in many aspects of RNA processing in organelles. The highly abundant gma-miR1508a/b (#22, Table 2) targets one subset of PPR genes including Glyma09g30500.1, Glyma16g27800.1, Glyma09g39260.1, and Glyma09g39250.1. On the other hand, our results suggest that gma-miR4413 (#23, Table 2) targets another subset of PPR genes including Glyma07g11410, Glyma07g11290, Glyma16g31950 and Glyma09g07250 and could be the inducer in the generation of the siRNAs shown to align to a broader region of Glyma09g07250 Exon 1 (Figure 5A). One of the features that differentiate siRNAs from miRNAs is their alignment to a broad region of the target gene, in contrast to miRNAs that generally align to only the 21- or 22-nt sequence matching the miRNA.

#### Auxin response factor

Bowtie analysis found over 400 unique smRNAs mapping to Glyma05g27580 that is annotated as an auxin response factor (ARF). These siRNAs aligned primarily to a 2 kb region at the 3’end of the 6 kb Glyma05g27580 gene (Figure 5B). In this instance, as was the case in the silencing of the *CHS* gene family [24], none of the auxin response factor siRNAs aligned to the predicted introns, indicating that the siRNAs were processed from spliced mRNA.

In addition, a second subgroup of 95 unique smRNAs annotated as Auxin response factor 6 mapped to a second gene, Glyma08g10550, with an auxin response factor motif. The smRNA signature with the most reads in this subgroup was found in the four seed coat libraries. The two gene models, Glyma08g10550 and Glyma05g27580, represent members of a gene family since their sequences have high degree of similarity. It is interesting that only the exons at the 3’-end of the two genes were the source of the two siRNA subgroups. A *G. max* BLAST search in Phytozome with the 2 kb region aligning to the siRNAs found two additional loci in Gm13 (Glyma13g29320) and Gm15 (Glyma15g09750) with an auxin response motif. However, only nine of the siRNAs in the auxin response factor subgroup aligned to Glyma13g29320, and one to Glyma15g09750. All four related genes map to four different chromosomes and it was not obvious where and how the primary inducer(s) of the auxin response factor siRNAs originated. Regardless of the place of origin, the pathway that generated them is tissue specific since all these siRNAs were found for the most part in the seed coat libraries. In sum, these two siRNA collections likely result from silencing at least two auxin response factor genes in a tissue specific manner in the seed coat.

#### Aquaporins

Another large group of approximately 400 unique smRNAs annotated as Aquaporin NIP6-1 mapped within or near a 5 kb genomic region in Gm14 containing an inverted repeat (621 bp) separated by approximately 1,000 nt of unknown sequence (Figure 5D). Two low confidence Glyma gene models (Glyma14g13250 and Glyma14g13260) with an aquaporin motif (MIP superfamily) were identified in the 5 kb region of the soybean genome containing the 621-nt sequence repeat. In addition, a BLAST search in Phytozome with the 621-nt sequence found similarity to several aquaporin Glyma gene models in chromosomes 7, 8 and 15. Alignments of those repeated regions to the smRNAs with the aquaporin annotation revealed that a region of exons 2, 3, and 4 in Glyma07g03030 that also matched the inverted repeat region on Gm14 aligned to 40 of the unique siRNAs with 0 mismatches (Figure 5E). The siRNAs representing this aquaporin siRNA group were found in all tissues examined, although the most reads were found in very young developing seeds, where the expression of the inverted repeat might be strongest with possible downregulation of the targeted aquaporin transcripts in the young seed.

In contrast, another group of smRNAs annotated as “Probable aquaporin TIP-type”, appear to be present only in the seed coats. Nearly 300 unique smRNAs mapped to chromosome 19 in a region identified as an aquaporin transporter (Glyma19g37000) while only 25 of these signatures mapped also to a chromosome 3 sequence encoding another aquaporin putative gene (Glyma03g34310). However, these aquaporin genes had no similarity to the inverted repeat found in Gm14 that created the primary siRNA inducers of the secondary siRNA group described above and thus, the biogenesis of these 300 seed coat tissue specific siRNAs with similarity to Glyma19g37000 and Glyma03g34310 remains unknown. Alignment of the siRNAs to Glyma19g37000 genomic sequence found them matching the two exons of this gene (Figure 5F).

#### Other putative siRNAs and their potential targets

Many of the annotations of other Glyma models that matched siRNAs predict their involvement in transcription or physiological processes, such as Glyma13g32810.1, annotated as MADS-box transcription factor 27 which could potentially translate a transcription factor involved in DNA-dependent transcription regulation. Also, Glyma09g02920.1, annotated as a “dsRNA-specific nuclease Dicer and related ribonucleases”, aligned to 37 unique siRNAs. A larger group of 334 unique siRNAs mapped to Glyma12g27800.1 annotated as a signal transduction transmembrane receptor with a TIR domain. *MultAlin*[40] clearly revealed they were dispersed siRNAs, some with high counts, aligned to this Glyma model which might silence this putative TIR disease resistance gene (Figure 5C).

### Small RNAs matching ribosomal RNA genes

**Figure 6:**
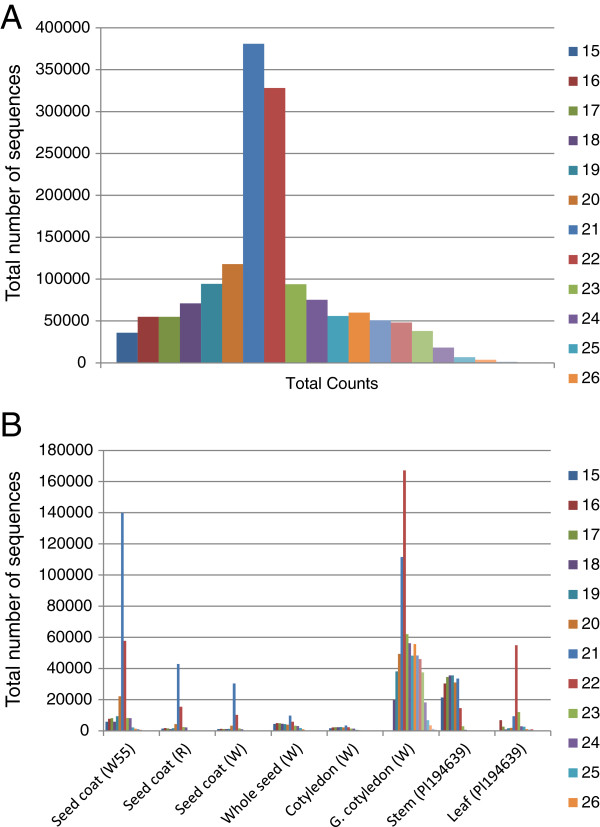
**Size distribution of all smRNA sequences aligning to chromosome Gm16 (33,069,649.33,072,849) genomic region containing the highly repeated 26S ribosomal RNA gene sequence.** (**A**) Total number of smRNAs without discriminating by tissue type. (**B**) SmRNA sequences in each one of the tissue/organ libraries studied.

We analyzed the small RNAs that mapped to more than 25 different locations in the soybean genome. One class of those siRNAs matched regions of the soybean genome on chromosomes 13, 15 and 16 that contain multiple repeats of 26S and 5.8S ribosomal RNAs. Our results showed that these regions were a source of siRNAs with a broad size range. These may be generated by different or multiple mechanisms. The sizes of the most abundant smRNAs generated by the 26S ribosomal repeated sequence were 21and 22 nts (Figure 6). We note that the current Phytozome soybean genome does not remove or annotate the ribosomal DNA regions correctly but shows them as having Glyma model numbers with varied annotations that predict peptide domains (see Discussion). The overall percentage of this ribosomal siRNA class ranged from a few percent in each library to a maximum of about 10% in the germinating cotyledon library.

### Small RNAs from transposons and retroelements

The most utilized small RNA pathway in plants appears to be the one involved in silencing repeated sequences including retroelements and transposons, and is dependent on the activities of RNA-dependent RNA polymerase (RDR2) and the Dicer-like protein (DCL3) to produce a large number of a highly diverse population of 24-nt siRNAs (Xie et al., 2004) [10]. These 24-nt siRNA sequences are affiliated with repeated sequences and function in association with argonaute protein (AGO4) and methyl transferase HEN1 to mediate transcriptional silencing of such repeated sequences by aiding DNA and histone methylation [10-12]. Current estimates indicate that up to 60% of the soybean genome consists of repeated sequences [41]. To identify and classify the sources of small RNAs from various repeat elements, Bowtie software was used against the soybean transposable elements database from Phytozome [33]. BLAST analysis of the 41,377 smRNA signatures 24-nt long against the soybean transposable elements database found that 5,187 (12.5%) matched repeated sequences with 95% fidelity. Interestingly, of all 107,279 smRNAs found to match the soybean genome database, 41,897 (39%) mapped to multiple (11 - 103) genomic locations. Of all the 24-nt smRNAs that found matches against the transposable element database (5,187) only 762 had more than 11 matches (from 11 - 51) to distinct genomic locations, suggesting that at least 6,767 additional 24-nt smRNAs that had more than 50 (50 - 103) matches in the soybean genome could be involved in regulating repeated sequences that are not represented in the currently available transposon elements database for the soybean [33]. Alternatively, many 24-nt smRNAs may be the result of silencing non-transposon related repeated sequences and non-repeated sequences.

**Figure 7:**
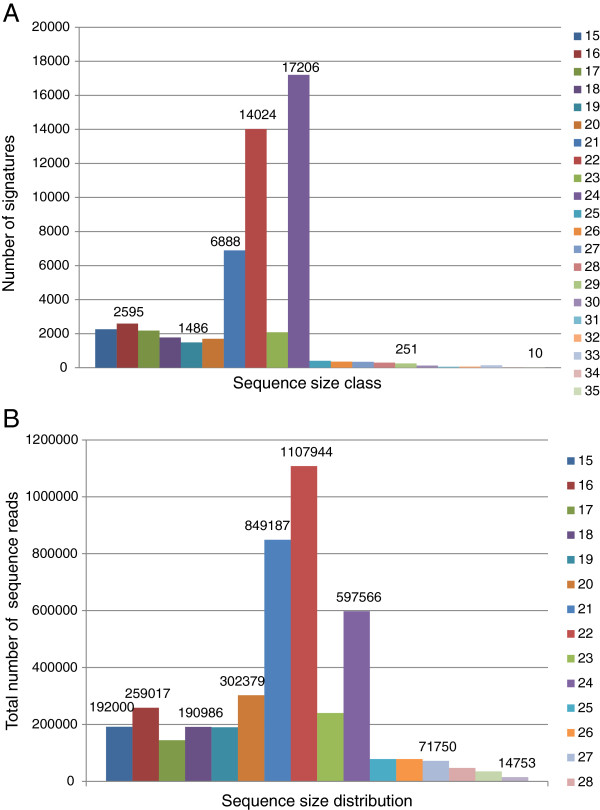
**Sequence size distribution of smRNAs targeting repeated elements in soybean tissues.** (**A**) Total number of unique signatures based on sequence size. (**B**) Total number of sequence reads in each size class. The numbers on top of the bars represent the number of signatures for some of the size classes.

An additional search using a different *G. max* (Williams 82) Transposable Element Database [42] was carried out to identify smRNAs from the entire collection that could be derived or linked to repeated elements and to a particular TE class. A total of 54,281 unique smRNA signatures matched sequences in this TE-database and of those, 27,731 were of the *Gypsy* family of elements, 11,397 *Mutator*, 11,165 *Copia*, 1,384 *PIF-Harbinger*, 1,377 L1, 553 *CACTA*, 311 unknown family, 170 *Helitron*, 76 *hAT*, 75 *Tc1-Mariner* and 42 *PONG*. Although the size range of the unique signatures associated with repeated elements varied from 15 to 35 nt, the 24-nt size class was the most abundant (17,206) followed by those with 22 nt in length (14,024) (Figure 7A). When the total number of sequences per signature was taken into account, the 22-nt size class was the most abundant (1,107,944) followed by the 21-nt (849,187) and 24-nt class (597,566) (Figure 7B) suggesting that the 24-nt signatures were more diverse than the 22 and 21-nt signatures. During the course of this analysis, it was observed that the extremely abundant soybean gma-miR3522 miRNA, previously mentioned to account for 3.9 million reads in the leaf library and 4.8 million total reads among all eight libraries, imprecisely matched a single position (Gm02:21389789.21394849) flanked by two LTRs and related to a *Gypsy* element. This 21-nt targeted site, with the 2-nt mismatches allowed in the Bowtie alignment, it is not repeated as such, in its entirety, elsewhere in the genome but rather in a degenerated form on ten other chromosomes. In these locations the imperfect 21-nt target site has been truncated separating the 16-nt at the 5’end from the 5-nt at the 3’end. If the *Gypsy* element on Gm02 were expressed, it is possible that it could be silenced by miR3522 despite the 2-nt mismatch within the 21-nt miR3522 sequence. However, we found no small RNAs complementing the Gm02 *Gypsy* element other than miR3522 in a bowtie search of all eight small RNA libraries indicating that the element is not likely expressed and silenced in the tissues examined. Regardless of the potential action of miR3522 on this family of retroelements, the high abundance of miR3522 is not tied to the repeated nature of the retroelement’s LTR ends and it’s sequence is even more degenerate in other locations and therefore, this extremely abundant miR3522 sequence was removed from the size distribution analysis of small RNAs matching to transposons shown in Figure 7.

**Figure 8:**
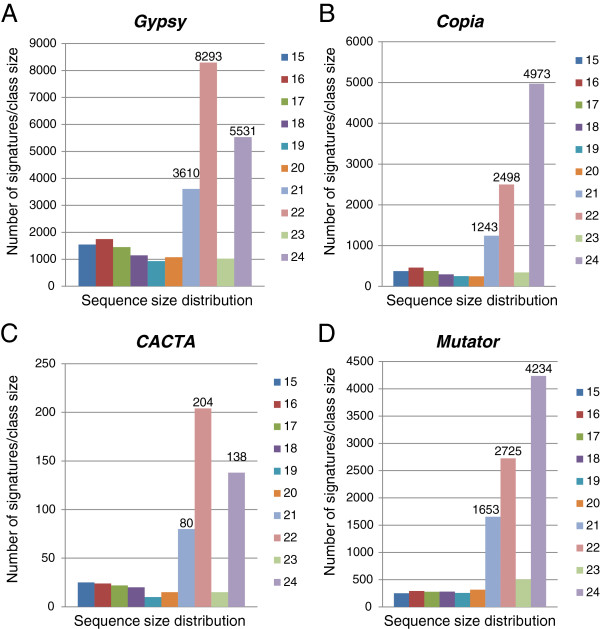
**Size distribution of unique smRNA signatures matching four different transposable element families. (A**) *Gypsy*. (**B**) *Copia*. (**C**) *CACTA*. (**D**) *Mutator.* The smRNA signatures with sizes above 24-nt were not included in this study since they were very few in comparison. The numbers on top of the bars represent the number of signatures for the most abundant size classes. The 22-nt signatures were the most abundant class for the *Gypsy* and *CACTA* families while the 24-nt were the most abundant for the *Copia* and *Mutator* families.

**Figure 9:**
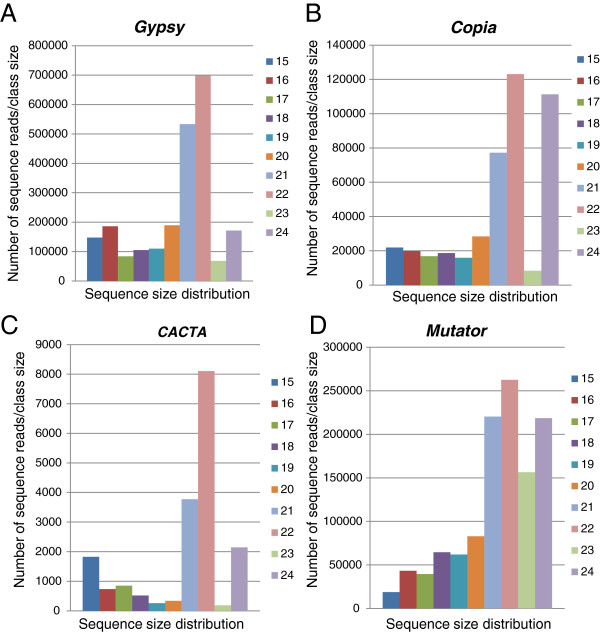
**Size distribution of total number of smRNA sequence reads related to four different transposable element families.** (**A**) *Gypsy*. (**B**) *Copia*. (**C**) *CACTA*. (**D**) *Mutator.* The number of total sequence reads per size class is shown. The 22-nt class had the most sequences in all TE families.

An intriguing observation was the distinct size preference of the number of unique siRNAs affiliated to each TE family as shown in Figure 8. This raised the possibility that the inactivation mechanisms of *Copia* (Figure 8B) and *Mutator* (Figure 8D) related sequences generate the 24 nt smRNAs predominantly, while the mechanism to inactivate *Gypsy* (Figure 8A) and *CACTA* (Figure 8C) elements produces 22 nt smRNAs predominantly. However, when the total number of reads per signature was taken into account, the 22-nt sequences outnumbered the 24-nt ones in all four TE families (Figure 9). Thus, the 22-nt smRNA class appears to be a relevant participant in the silencing of most TE families in soybean.

### *Glycine max* putative trans-acting siRNAs and *TAS3* related genes

In *Arabidopsis* miR173, miR390 and miR828, promote the processing of a specific class of small RNAs, known as trans-acting small interfering RNAs (tasiRNAs), which in turn target other mRNAs for post-transcriptional downregulation. Four families of *TAS* genes have been demonstrated in *Arabidopsis* to date: miR173 triggers the processing of tasiRNAs from *TAS1* and *TAS2* genes, miR390 from *TAS3*, and miR828 from *TAS4*[14-16,43]. A characteristic of tasiRNAs processing is that the cuts of the *TAS* mRNAs are phased, in 21-nt intervals, from the start at the miRNA binding site [16].

**Figure 10:**
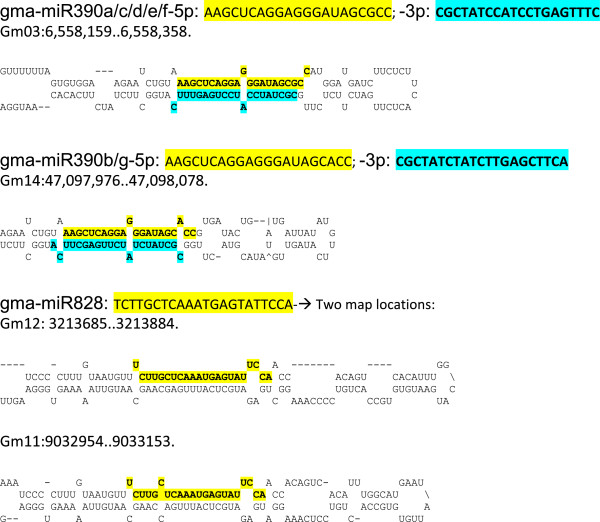
**Stem loop secondary structures of gma-miR390 and gma-miR828 RNA precursors.** The miR390 targets the *TAS3* gene and miR828 targets the *TAS4* gene in *Arabidopsis.* The miR390b-3p (CGCTATCTATCTTGAGCTTCA) found in our smRNA collection differs with respect to that entered in miRBase with Accession number MIMAT0020935.

In soybean, no miR173 or miR828 sequences have been entered in miRBase to date. A search of our entire smRNA populations found no sequences related to miR173, which appears to be unique to *Arabidopsis*. However, we identified two putative miR828 loci (Gm12:3213774. 3213795 and Gm11:9033043. 9033064, Figure 10) as well as a putative *TAS4* gene (Glyma19g44660) based on sequence similarity to the *Arabidopsis* counterparts. Very low expression of miR828 was found only in seed coat smRNA libraries (Table 3) and no tasiRNAs derived from the putative *TAS4* gene were found. In contrast, two miR390 genes have been entered in miRBase, mapping to Gm3 (miR390a) and Gm14 (miR390b). In addition, we found five additional loci able to form stable secondary structures using RNA-*mfold,* four of which may encode miR390a-like miRNAs and another one encoding a miR390b-like sequence (Figure 10). Table 3 shows that the sequence count level of gma-miRNA390a and gma-miRNA390b were several hundred in many of the tissue libraries.

**Table 3:** **Differential expression of soybean miRNAs that target*TAS3*and*TAS4*Gene Homologs in Soybean**

			**Normalized counts in each library per million reads**^**a**^										
**Sequence**	**L**	**miRBase**	**Immature seed tissues**					**Vegetative tissue**			**miRNA gene origins**^**b**^	***Putative TAS* gene**^**c**^	**tasiRNA target and annotation**
			**WS**	**SCR**	**SCM**	**SCW**	**Cot**	**GCot**	**ST**	**LE**			
AAGCTCAGGAG GGATAGCGCC	21	gma-miR390 a/d/e/f-5p	456	116	166	113	275	132	20	193	Gm18: 5,047,835	Glyma09g03730.1	Glyma07g32300.1 AUX-IAA-ARF
											Gm18: 53,278,046	Glyma15g14670.1	Glyma13g24240.2 AUX-IAA-ARF
											Gm11: 30,272,772		
											Gm03: 6,558,249		
											Gm01: 42,335,625		
AAGCTCAGGAGGG ATAGCACC	21	gma-miR390b/ g-5p	24	6	2	2	153	60	165	12	Gm14: 47,097,986	Glyma09g03730.1	Glyma07g32300.1 AUX-IAA-ARF
											Gm12: 44,954,768	Glyma15g14670.1	Glyma13g24240.2 AUX-IAA-ARF
TCTTGCTCAAATGA GTATTCCA	22	gma-miR828	0	7	6	7	0	0	0	0	Gm12: 3,213,774	Glyma20g32510.1	
											Gm11: 9,033,043	Glyma10g35050.1	
												Glyma20g32500.1	
												Glyma09g37340.1	

^a^Library abbreviations are in Table 1. ^b^The position of the miRNA hairpin in the *Glycine max* reference genome is shown. ^c^The gene model (Glyma number) of the putative *TAS* genes in soybean.

L = sequence length.

**Figure 11:**
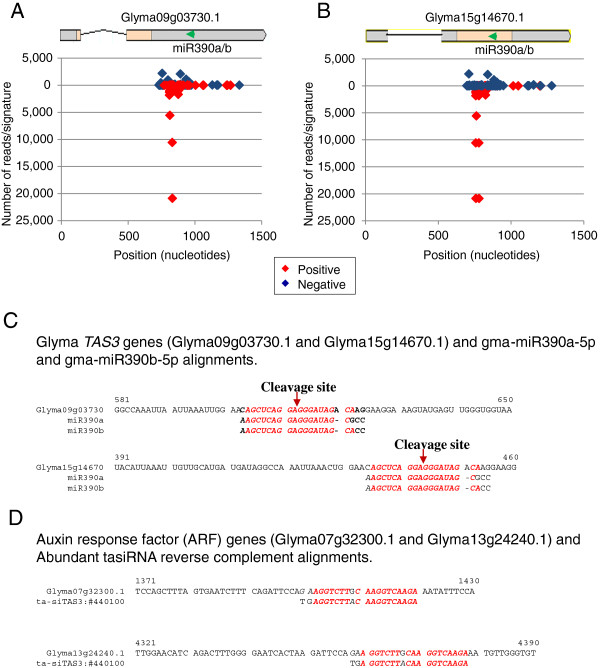
**Alignment of tasiRNAs to putative soybean *TAS3 *genes Glyma09g03730.1 and Glyma15g14670.1.** The tasiRNA signatures aligning to Glyma09g03730.1 (**A**) and Glyma15g14670.1 (**B**) genomic sequences with 100% accuracy were sorted out from the Bowtie search. Both genes are related although their predicted structures differ. (**C**) Alignment of miR390a/b to Glyma09g03730.1 and Glyma15g14670.1. (**D**) Alignment of the most abundant tasiRNAs to two ARF genes, Glyma07g32300.1 and Glyma13g24240.1. Green arrow in (A) and (B) mark the binding site of gma-miR390a/b.

The expression level of at least two miR390 family member genes with hundreds of reads suggested the likelihood of these miRNAs targeting an ortholog of the *Arabidopsis TAS3* gene. A nucleotide-BLAST search in Phytozome using the *AthTAS3* genomic sequence (NR_022742 = gb|CP002686.1: 5861491-5862437) identified two loci on *Glycine max* Gm9 and Gm15. Both genes coincided with two predicted low confidence gene models with 87% identity, Glyma09g03730 and Glyma15g14670, indicating the existence of at least two putative *TAS3* genes in *Glycine max*. Alignment of miR390a-5p and gma-miR390b-5p signatures to both *TAS3* genes found that they aligned to the same region in both genes with 17 and 18 identical matches, respectively (Figure 11C). These results suggest that both miRNAs could target transcripts from the two *TAS3* genes and mediate a breakage. The fractured mRNA product would then be converted to double-stranded RNA by the activity of RNA-dependent RNA polymerase 6 (RDR6) followed by Dicer-like 4 (DCL4)-mediated cuts to process phased tasiRNAs.

To determine the existence and nature of tasiRNAs derived from these putative *TAS3* transcripts, the Glyma09g03730.1 genomic sequence was used as the reference in a Bowtie search of the soybean smRNAs. A collection of 218 tasiRNA unique signatures with no mismatches that aligned to the Glyma09g03730.1 genomic sequence in phased, 21-nt intervals, were recovered (Figure 11A and 11B and Additional file 3). The tasiRNA signatures with the most reads were found in the stem at 2,999 and 1,593 normalized reads. All tasiRNAs aligned to the 3’UTR of the low confidence Glyma09g03730.1 gene model. The two putative *TAS3* genes have similar sequences but different predicted structures. The tasiRNAs were generated from the 3’UTR region of Glyma09g03730.1 (Figure 11A), but they may derive from an exon and 3’UTR in Glyma15g14670.1 (Figure 11B).

In *Arabidopsis*, the *TAS3* tasiRNAs regulate leaf patterning, developmental timing and rate of lateral root growth by repressing the Auxin Response Factors (ARF- 2, - 3 and -4) (Marin et al., [2010]). To identify putative *Glycine max* ARF target genes for the most abundant *TAS3* tasiRNAs*,* alignments to 12 Glyma model sequences annotated in Phytozome as auxin response factors *(*ARF) were examined. The transcripts with the most nucleotide matches (18) at the tasiRNA target site were Glyma07g32300.1 and Glyma13g24240.2 (Figure 11D). These results in turn predict that these putative *Glycine max* ARF genes may be regulated by a complex mechanism as it occurs in *Arabidopsis.*

## Discussion

### Variation in soybean miRNA genes and their expression in seed and vegetative tissues

A comprehensive analysis was compiled on variation in the miRNAs present in a large population of 41 million smRNA sequences derived from eight libraries representing multiple tissues/organs of the soybean plant. At least 4,031 unique signatures corresponding to 213 miRNAs and representing 117 families were detailed for each of the sequences falling within each family ( Additional file 2). Examination of the number of reads of each signature revealed all the length or sequence variations found in the high throughput populations for all miRNA family members. Thus, the flux of expression patterns for each of the unique signatures in eight libraries representing both seed and vegetative tissues of the seedling are detailed. Preferential expression in the seed coats or in cotyledons of immature seeds as shown for certain family members by sequencing were verified by small RNA blots (Table 2, Figures 2 and 3). Some of the miRNAs were very abundant in certain tissues such as members of the miR167 family and gma-1512c, which were highly prevalent in the seed coats from multiple samples. Several others showed preferential expression in either of the germinating cotyledons, stems, or leaves. One single miRNA, gma-miR3522, accounted for 11% of the total reads in the immature cotyledons and an astonishing 65% of the total reads in the 1-month-old expanded leaf library. Promoter analysis of the gene (Glyma15g06080) encoding miR3522 found it contained cis-acting regulatory elements for tissue specific and enhanced gene expression ( Additional file 4). In the leaf sample, the diversity of unique small RNAs was reduced by four to five-fold compared to all the other libraries (Table 1). Whether this resulted from preferential amplification of the highly abundant gma-miR3522 during the cluster formation in the sequencing reaction is unknown. However, all of the other library samples yielded 27-38,000 diverse sequences and yet some individual miRNAs signatures repeatedly accounted 1-5% of the total counts in the multiple seed coat libraries. Thus, the high count miRNAs are likely biological in nature.

Of particular interest was the distinct tissue expression of two subgroups of the same miR156 family. Although the sequences of the two subgroups differed only in one nucleotide, the gma-miR156c/d/e/g members were expressed at very low levels in the developing cotyledon (25 counts) while gma-miR156b/f members were expressed at high levels to 98,674 normalized counts which was nearly 10% of the same smRNA population of the cotyledon sample. The reverse is true for the stem and leaf expression (Table 2). The gma-miR156c/d/e/g signature found matches to at least seven loci in six chromosomes with coding potential for miR156 genes. Given the low number of reads (25) in the developing cotyledon, one possibility is that many of these seven miRNA genes are suppressed in a tissue specific manner. The coordinated regulation of so many miRNA genes dispersed through at least six chromosomes could involve a transcription factor [44]. In all, the miR156 gene family in soybean is composed of at least 18 putative member genes expressed differentially. The mature miR156 family members have been shown to target and silence genes of the SBP transcription factor family in *Arabidopsis*[38]. Our results show the power of high throughput sequencing to quantitatively distinguish the differential expression of miRNA family members with single nucleotide polymorphisms in the mature miRNA sequences, which is not possible to do with blotting and difficult to achieve with quantitative RT-PCR.

In summary, from the selection of soybean miRNA sequences in Table 2 and Additional file 2, it is clear that tissue differences in normalized read counts are apparent in many of the known miRNAs that are conserved widely in plant systems as well as some that were more recently found to be soybean specific [28-31]. Additionally, by mapping the origins of the soybean miRNAs from our samples to genomic locations that are capable of forming stable secondary RNA hairpin structures, we identified 31 new family members of various soybean miRNAs. Based on sequence similarity many possible targets were also identified for the soybean miRNAs.

### Differential expression of miRNAs at the 5’ and 3’ end of miRNA precursors

Some of the miRNA genes were observed to encode two miRNAs at the 5’ and 3’ ends of the RNA hairpin precursor. We document three instances in which the miRNA signatures derived from the 3’end (3p) were present in higher or lower abundance than those from the 5’ end (5p).

Three such examples are illustrated in Table 2 and Figure 4: miR166a (#10, #11), miR1510a (#14, #15) and miR1510b (#13, #15). The clearly distinct sequences at the two ends of the stem loops overlap by 2 nt in all these cases and accumulated at very different levels. Sometimes the 3p-miRNA accumulated at higher level than the 5p-miRNA (miR166a), but the opposite was observed as well like in the case of miR1510a/b. Whether the miRNA processed on the 3p side of the hairpin is induced under biotic stress as was found for some of the miRNAs examined in *Arabidopsis*[45] is unknown, but both of these gma-1510a/b-3p miRNAs potentially target proteins involved in disease resistance pathways as predicted by sequence matches (Table 2). Another example not shown in Table 2 but expressed in a similar way was gma-miR482-5p versus its -3p counterpart.

### Identifying genes that may be silenced based on recovered siRNAs

Whereas miRNA genes are defined by a small hairpin structure in the genome, the origin of siRNAs can be less obvious but is often associated with larger inverted duplications that may spur production of dsRNA or antisense RNA. Alternatively, they may result from miRNA/siRNA directed transcript cleavage followed by RDRP synthesis of longer dsRNA from the fractured and siRNA segments encompassing a broader region of the transcript. One of the key differences that aids in distinguishing miRNAs and siRNAs is the distribution of the alignment of smRNAs with the sequence of the genes from where they are derived. The miRNAs typically align to a ~ 21-nt region of the gene sequence with a preferential bias in the number of copies representing one strand versus the opposite strand, and thus, it result in relatively few different signatures. In the case of siRNAs, there is an extended region of the sequence aligning with perfect or near perfect match to many diverse signatures that cover both strands of a putative silenced gene, although only smRNAs complementary to the mRNA strand would be effective in the gene’s continued downregulation.

Although much is known about the miRNAs in plants, relatively little is known about the siRNAs. One clear example of siRNAs that alter a plant phenotype are soybean *CHS* siRNAs that have been shown to downregulate the *CHS* gene family leading to reduced pigment in the yellow seed coats of soybean lines with dominant *I* or *i*^*i*^ alleles at the *I* locus [24]. The origin of the *CHS* siRNAs is an inverted repeat cluster of *CHS* genes and not a miRNA. Mutations to pigmented seed were shown to be deletions in the repeat cluster region that negate the production of dsRNA and *CHS* siRNAs resulting in CHS enzymatic activity and pigmented seed coat.

The pattern of alignment of the smRNAs to potential Glyma model transcripts is one way to distinguish siRNAs and miRNAs. Following the example of *CHS* siRNAs [24], we looked for smRNAs that aligned to a broader region of a Glyma model than typical for a miRNA. Assortment of the smRNAs by annotation groups enabled the discovery of putatively silenced genes and in some instances the miRNA or siRNA origins that may trigger silencing. Among these were signatures annotated as PPR repeats, analysis of which revealed the expression of three miRNA genes that target several PPR repeat gene families. Sequences miR1508a and miR1508b (#22 in Table 2) were expressed in all tissues but with preference in germinating cotyledons with a normalized count of 7,892 reads per million. They target a set of PPR genes including Glyma09g30500, Glyma16g27800, Glyma09g39260 and Glyma09g39250. On the other hand, miR4413 (#23 in Table 2) had the potential to target a different family of at least six PPR genes including Glyma09g07250. Additionally, we show that miR4413 is the inducer of a collection of siRNAs shown to originate from exon 1 of Glyma09g07250.1 (Figure 5A). Our results from the *Multalin* sequence alignments for the Glyma genes and derived siRNAs in Figure 5 show the same distribution pattern as those obtained with the *G. max TAS* genes and gma-tasiRNAs. We can then infer that the processing of siRNAs such as those from the PPR gene (Glyma09g07250.1) is phased as well.

Two other potentially silenced genes, Glyma05g27580 (Figure 5B) and Glyma08g10550 (not shown), both encoding putative auxin response factors, were revealed by two separate smRNA sets. Since additional related genomic sequences reside in separate chromosomes it was difficult to decipher the driving mechanism that could induce the production of siRNAs matching those two types of auxin response factor genes. However, it appears to be tissue specific since the majority of the siRNAs were found in seed coat libraries. This could be another example of silencing specific to the seed coat as that exhibited by the abundant *CHS* siRNAs [24]. Glyma12g27800, encoding a disease resistance protein containing a TIR-motif, was identified as the source of a collection of 334 unique siRNAs (Figure 5C). Other genes, Glyma13g32810 and Glyma15g06490, annotated as “Mads-box transcription factor 27” were found to be the likely source of a collection of 77 unique siRNAs.

In one case, both the smRNA origin and the potentially silenced genes were identified for a specific family of aquaporin genes. The Glyma07g03030 gene annotated as aquaporin NIP6-1 (Figure 5E) could be targeted by at least 41 primary siRNAs originating from the inverted repeat found on Gm14 (Figure 5D). The aquaporin siRNAs derived from the inverted repeat and Glyma07g03030 were found in all tissues examined suggesting that they are constitutively generated and able to target this aquaporin family member gene. In contrast, a second group of aquaporin transporter siRNAs resulted from Glyma19g37000 (Figure 5F) and Glyma03g34310 genes and they are not induced by the primary siRNAs originating from the inverted repeat in Gm14 (Figure 5D), as there is little sequence similarity between the aquaporin repeat and the Glyma19g3700 and Glyma03g34310 aquaporin transporter genes. The mechanism that induces the production of this second aquaporin siRNA group remains undetermined but it appears to be limited to the seed coats based on the tissue specific occurrence of the siRNAs aligning to Glyma19g37000.1 (Figure 5F).

### Potential silencing of highly repeated genomic loci including ribosomal genes and transposons

During investigation of the nature of smRNAs that match to more than 10 positions in the soybean genome, we discovered that many of these were siRNAs derived from ribosomal RNA genes (rDNA) representing the 26S rRNA sequence (AK286061.1) and 18S rRNA gene (X02623.1) although the annotations of these genomic regions were not shown as rDNA genes in Phytozome. Instead, Phytozome has assigned Glyma model numbers and peptide motifs to these coding regions including a serine-threonine protein kinase gene (Glyma16g29220) with similarity to only the 26S rRNA sequence (AK286061.1) and several predicted genes such as allatostatin (Glyma13g11820), polymerase Rpb1 C-terminal repeat (Glyma13g11940) and copper binding octapeptide (Glyma13g11980). These occur in up to 14 repeated sequence stretches containing the 26S rRNA (AK286061.1), the 18S rRNA (X02623.1) or 5.8S rRNA (FJ980442.1) genes. Recently, a study [46] showed that misannotations of rDNA genes can generate false positive matches in many sequencing studies of both prokaryotes and eukaryotes as these misannotations have been propagated in databases such as GenBank and SwissProt. The more than 15 Glyma models in Gm13 containing multiple rRNA repeats may constitute the nuclear organizer which may protect the ribosomal transcripts from silencing. The siRNAs that complement these ribosomal gene sequences may derive from similar sequences repeated in other chromosomes such as the above mentioned 26S rRNA insertion in Glyma16g29220 (a serine-threonine protein kinase gene) also found in Gm05, Gm15, Gm14 and several others.

The number of ribosomal siRNA sequences found in the eight libraries ranged from a few percent to approximately 10% in the germinating cotyledon library. Whether the fluctuation in the abundance of these siRNAs reflects a physiological silencing of certain ribosomal genes in tissues in which the ribosomal related siRNAs are highest is unknown. However, the size distribution indicates these are authentic small RNAs and not rRNA degradation products since the smRNA sizes peak in the 21 to 22-nt range as shown in Figure 6. The same thing could be said for the siRNAs aligning to the putatively silenced genes shown in Figure 5.

The global analysis of smRNAs matching repeated sequences was advanced to include available soybean transposable element databases. The distinct size preference of smRNAs resulting from different TE families suggested the possibility of diverse interaction mechanisms for *Copia* (Figure 8B) and *Mutator* (Figure 8D) in which the 24-nt signatures dominated, versus the *Gypsy* (Figure 8A) and *CACTA* (Figure 8C) families in which the 22-nt signatures were most abundant. This difference was blurred when the total number of reads per signature was taken into account, in that all of the TE families had the 22-nt signatures as the most abundant (Figure 9). Thus, the 22-nt smRNAs seem to be a major component in the silencing of most TE families and repeated sequences in soybean.

Interestingly, in a global smRNA size distribution analysis we found that the 24-nt smRNAs were the most prominent class in the seed (immature coat and cotyledon) libraries while in the organs from germinating or adult plants (stem, leaf and germinating cotyledon) the 21-nt smRNAs had the largest number of unique reads (Figure 1A and B). Second in abundance in the seed smRNA libraries were the 21 and the 22-nt smRNAs. However, for the very young whole seed and cotyledon smRNA libraries the 22-nt unique signatures were more abundant than the 21-nt ones (Figure 1A and B). It has been proposed that the 22-nt smRNAs are required as guides of AGO/DCL/RDR2 complexes to drive the second RNA strand synthesis substrate of phased secondary siRNAs [19]. However, this size class could be the result of other procedures and serve other functions. In maize, a significant number of 22-nt siRNAs were found in root and shoot tissues that appear to function differently from the 21- and 24-nt siRNAs based on their origin and targeting region of genes [4]. In the soybean seed coat smRNA populations, the 22-nt smRNAs were as abundant as the 21-nt, suggesting a relevant role of the 22-nt smRNA in seed coats as well.

Why the 24-nt smRNAs are the most abundant class of unique sequences in seed tissues compared to germinating cotyledons and the vegetative stem and leaf tissues is intriguing. It may be an indication that repeated sequences such as transposable element sequences are actively transcribed in the developing seed tissues which serve as substrates for siRNA generation which in turn will mediate gene silencing of the repeated sequences through DNA/histone methylation. In *Arabidopsis,* silencing of transposable elements in the embryo is enhanced by an abundance of mobile 24nt-siRNAs generated through the reactivation of transposable elements in the seed endosperm [47-49]. Note that the highest numbers of diverse 24-nt smRNAs (50% of the total number of unique sequences) in the soybean libraries were found in the very young seed (12-14 DAF) and the immature cotyledons (Figure 1A and B).

As in the soybean seed tissues where the 24-nt class is highest, in *Arabidopsis* wild type (Col-0) inflorescence tissues, the number of 24-nt siRNAs was found to be 3 and 10 times higher than the 21-nt size class [1,10]. Likewise, in maize immature ears, the number of 24-nt smRNAs was found to be 2.5 times higher than the 21-nt size class [25]. In contrast to the soybean stem and leaf results where the 21-nt signatures were more abundant than the 24-nt smRNAs, in maize shoots and *Arabidopsis* leaves, the 24-nt smRNAs outnumbered the 21-nt smRNAs [4]. However, in *Arabidopsis* seedlings, the 21-nt class was slightly more abundant than the 24-nt smRNAs, resembling the size distribution in the soybean stem [1].

### Identification of *Glycine max* trans-acting siRNAs and *TAS* genes

The search for miRNAs that could direct the biogenesis of putative trans-acting siRNAs from *TAS* gene orthologs in soybean identified two members of the miR390 family. These gma-miR390s are also likely inducers of tasiRNAs from two putative *G. max TAS3* genes, Glyma09g03730 and Glyma15g14670 that had 87% identity with *AthTAS3*. Evidence of this process occurring in soybean was the existence of a collection of 397 tasiRNA signatures that aligned to the two putative soybean *TAS3* genes. In addition, at least two *Glycine max* ARF genes, Glyma07g32300 and Glyma16g02650 were chosen as likely targets for the most abundant *TAS3*-derived tasiRNAs (Figure 11D). These results predict that several putative *Glycine max* ARF genes may be regulated by a miR390/*TAS3*-derived tasiRNAs mechanism as it occurs in *Arabidopsis* where the *TAS3* tasiRNAs regulate leaf patterning, developmental timing and rate of lateral root growth by repressing ARF2, 3 and -4 genes. The positive and negative feedback regulation of miR390 by auxin signaling factors maintains ARF expression at a concentration that will determine the developmental timing of various organs formation [50]. Because in *Arabidopsis* cleavage of *TAS3* is unique in that it requires the specific action of the miR390/ARGONAUTE7 (AGO7) complex for tasiRNA production [51], our results then may anticipate the existence of an AGO7-like function in soybean.

## Conclusions

A global landscape of the non-coding smRNAs existing in the soybean plant growing under optimal conditions was revealed from the analysis of eight smRNA sequence populations with high throughput Illumina sequencing. The eight smRNA sequence populations representing five tissues/organs of the soybean amounted to a total of 41 million raw sequences that collapsed into 135,055 unique reads. All unique sequence reads were classified and characterized based on their abundance in the tissues/organs of origin, genomic origin, and possible target genes. Full analysis revealed 117 soybean miRNAs and 213 family members from the 4013 unique miRNA sequences. Some of the miRNAs were in very high abundances. Blots of smRNAs using oligo probes representing the reverse complement of the smRNA sequences confirmed the expression differences for some miRNAs within different immature seed or vegetative tissues. Predicted target genes for miRNAs, including members of the miR156 family whose family members showed highly differential expression, were analyzed. Some of the sequences processed into siRNAs were determined to be auxin response factors and aquaporins that may have important roles during seed development. Based on homology to an *Arabidopsis TAS3* gene, two soybean equivalent *TAS3* orthologs were identified and found to be targeted by the gma-miRNA390a/b family members. After miRNA guided cleavage of the soybean *TAS3* transcripts, phased 21-nucleotide tasiRNAs are processed that, in turn, could target genes with auxin response factor motifs.

Differences among the smRNA classes of immature seed versus vegetative tissues were noted with the seed tissues having significantly higher percentages of the 24-nt size class likely, representing siRNAs from silenced repetitive regions. Among the smRNAs associated with repeated sequences including rDNAs and transposons, the 22-nt smRNAs were also a highly abundant class, indicating their relevant role in silencing sequence repeats in soybean.

## Methods

### Plant materials and genetic nomenclature

The *Glycine max* cultivars used for this study were obtained from the United States Department of Agriculture Soybean Germplasm Collections (Department of Crop Sciences, USDA/ARS University of Illinois; Urbana, IL). The genotypes of the lines are described in Table 1. All lines are homozygous for the loci indicated and only one of the alleles is shown for brevity.

Plants were grown in the greenhouse and tissues harvested from at least four plants of each line. Leaves and stems were harvested from two to four-week-old plants. Stem samples consisted of one centimeter size fragments immediately underneath the base of the unifoliate leaves. The leaf sample was made of first trifoliate sampled over the course of one week. Germinating cotyledons were harvested from two weeks old seedling. Seed coats and cotyledons were dissected from seeds at varying stages of development based on the fresh weight of the entire seed. Dissected seed coats and cotyledons from seeds of the 50-75 mg weight range were used for the present study. The very young whole seeds were collected 12-14 days after flowering. Flowers were tagged on the first day they were fully opened (Day 0) and were tracked to the desired developmental stage. The young pods were harvested 12-14 days later and the whole seeds dissected out under an Olympus SZ61 microscope (Melville, NY). Whole young seeds were placed in microfuge tubes kept on ice during harvest and dissection. The collected very young whole seeds were fast frozen in liquid nitrogen upon collection and stored at -80°C until further use.

### Total and small RNA extraction

The RNA from very young whole seeds at the chosen developmental stage (12-14 days after flowering) was extracted using a modified version of the McCarty [52] procedure. The fast-frozen seeds were finely ground using a FastPrep FP120 (Savant Instruments, Holbrook, NY) for 20-30 seconds. Immediately after, 350 μl of Complete RNA Extraction Buffer (0.1 M Tris-HCl; 0.2 M NaCl; 20 mM EDTA; 10 mM dithiothreitol; 16 mM mercaptobenzothiazol; 2% Sarkosyl; 15 mM mercaptoethanol) was added to each sample and total RNA extracted using phenol-chloroform and lithium chloride precipitation methods [53,54].

For the seed coat, cotyledon, leaf and stem samples total nucleic acids were extracted using the standard phenol chloroform method [54] or TRIzol reagent (Invitrogen, Carlsbad, CA, USA) method [55]. Seed coats and cotyledons of Williams 55 line were pretreated using Wang et al [53] protocol prior to total nucleic acids extraction. For seed coat and cotyledon samples low molecular weight (LMW) RNAs were isolated as described previously [56] with minor modifications [24].

### RNA extraction for gel blot analysis

For the small RNA gel blots reported here total RNAs were isolated using our standard protocols [54] with the exception of the LiCl precipitation step that was eliminated. It is worth noting that such a protocol utilizes lyophilized tissues stored at -20°C and as the results show the small RNAs were well preserved.

Total RNA (50 μg) concentrated in 20 μl 25% formamide was denatured at 70°C for 10 minutes. Denatured RNAs were fractionated on 15% polyacrylamide 7 M urea denaturing gels, then transferred to Hybond-NX (or N) membrane (Amersham, Piscataway, NJ) using a Bio-Rad Trans-Blot apparatus (Bio-Rad, Hercules, CA) at 100 V for 1 hour. The membranes were dried and UV-crosslinked (Stratalinker, Stratagene, La Jolla, CA). Prehybridization was carried out in 50% formamide, 7% SDS, 0.05 M NaHPO_4_/NaH_2_PO_4_, pH 7.0, 0.3 M NaCl, 5X Denhardt’s solution and 100 μg/ml sheared denatured salmon sperm DNA at 40°C for at least 2 hours. Hybridization was performed in the same solution by adding the denatured [γ-^32^P] ATP labeled oligoprobe at 40°C for 15-20 hours. The filters were washed in 2X SSC and 0.2% SDS at 40°C for 15 minutes and exposed to Hyperfilm (Amersham, Piscataway, NJ) with enhancer screen overnight or 24 hours.

Small RNA reverse complement DNA oligoprobes were T4 Polynucleotide Kinase (PNK) 5′ end-labeled with [γ-^32^P] ATP using the Promega 5’ end labeling kit (*Cat.#* UD2010). Labeled oligoprobes were denatured for 10 min at 70°C prior to hybridization. Filters were prehybridized for at least 2 hr at 40°C prior to hybridization.

For accurate sizing of the siRNA species, an RNA ladder (10 – 150 nt) was used that had been radiolabeled with [γ-^32^P] ATP following the protocol provided with the Decade™ Markers Kit from Ambion, Inc. (Austin, TX).

### Sequencing of small RNA libraries and data analysis processing

Gel purification, cloning and sequencing of small RNAs from the multiple tissue samples was performed at Illumina Inc. (San Diego, CA) using the SBS (sequencing by synthesis) technology. Briefly, 2.5 – 5 μg of the purified LMW RNA fraction (or total RNA) of each sample was provided to Illumina, which for subsequent quality checks, was separated on 15% polyacrylamide gels containing 7 M urea in TBE buffer (45 mM Tris-borate, pH 8.0, and 1.0 mM EDTA). A gel slice containing RNAs of 15 to 35 nucleotides was excised and eluted. Gel-purified small RNAs were ligated to the 3’ adapter (TCGTATGCCGTCTTCTGCTTG), and the resulting small RNA libraries were sequenced using the Illumina Genetic Analyzer. Sequence information was extracted from the image files with the Illumina Firecrest and Bustard applications.

A total of 40,814,540 reads that were 35 bases long were obtained from the above libraries deep sequencing. After removing artefacts (products of multiple adapter ligations or empty constructs), 244,994 sequences remained for analysis. Adapter trimming was performed using the substring TCG as the unique identifier for the beginning of the adapter (TCGTATGCCGTCTTCTGCTTG). The sizes of the small RNAs after adapter trimming ranged from 15-35-nt with 20% having 21- nt.

The adapter trimmed sequences were then subjected to a script count.pl* that added up identical sequences to produce a merged fasta file containing the raw counts for each unique sequence from a given library. Sequences with a count smaller than *k* =5 (or 10 or 16) were removed as possible artifacts and to reduce the computational cost for subsequent steps. *k* was set to 5 in the earlier sequenced libraries with total read count of < =3 million (M) reads, set to 10 for libraries with 6 M reads and 16 for the latest libraries with >=12 M reads. After processing each of the libraries in this manner the sequences were imported into a database in a non-redundant manner i.e., a sequence is present only once in the database irrespective of the number of libraries that has it. This database stored separately the number of reads for each sequence in each library. The threshold for the presence of any sequence in the database was to be represented by a minimum of *k* reads in at least one library. A total of 135,055 unique small RNA sequences were thus identified from the eight libraries in this study.

### Annotation

Each sequence from our database was used in BLAST searches against NCBI nucleotide (nt) [36] and miRBase [35] databases. In both cases the BLAST output was parsed to identify alignments that had no mismatches in a match length of 18 bases or less and one mismatch in longer alignments. Annotations, if any, were then uploaded to the database. In addition each sequence was aligned against the soybean genome (*Glycine max*: Glyma1), using Novoalign [34]. The alignment did not limit the number of mismatches allowed, but the most seen was 4, indicating that mapping quality is too low for anything with higher mismatches.

At this stage a column-delimited text file listing the internal database ID, sequence and its length, annotation for all unique small RNAs and the number of reads from each of the libraries was exported from the database. Also appended at this stage was a column for the genomic location(s) this sequence maps. In cases where the sequence mapped to multiple locations, only those sequences mapping to less than 10 regions were entered. In parallel a script called addModel.pl* creates a separate file with the sequence ID, genomic location(s) and any gene models (Glyma Models) that are anchored in those locations. The GFF file from Phytozome was used to retrieve the predicted gene model, if any, at the mapped location. The gene model information was fed back into the database to enable easier retrieval in the future. Annotation.pl* added annotation information of the gene model to the file. A final script called teratogen.pl* consolidated the two files: column-delimited file with counts and the genomic locations and annotation file, using the sequence ID to generate the AllSoyExpanded Excel spreadsheet.

The content of the resulting Excel file was used for all subsequent smRNA data comparisons. Alignments of these curated smRNA sequences to one or multiple genomic DNA sequences were performed using BLAST in Phytozome [33] and Bowtie [39]. The results from these analyses were further characterized, cross compared and scrutinized with Excel table’s tools and in-home scripts. In a few instances detailed alignments were performed with *MultAlin*[40,57].

### Novoalign

Novoalign developed by Novocraft [34] was used to identify the origin of the small RNA in the soybean genome. Using the whole genome as reference instead of the smaller CDS sequences allows identification of small RNA generating loci without applying the slightly arbitrary limitation of computationally identified genic and/or repeat locations. Glyma 1 version of the soybean genome was used as reference.

### Accession numbers of small RNA data sets

Small RNA data sets (Illumina SBS) used in the study are available through the Gene Expression Omnibus (GEO) database [58]: Whole seed W (12-14 DAF) (GSM899820); Seed coat, Richland, (GSM543393); Seed coat W55 (GSM543396); Seed coat W (GSM543394); Cotyledon W (GSM543395); G. Cotyledon W, 7 day seedling, (GSM899821); Stem, 2 week plants, (GSM899822); Leaf, 1 month plants, (GSM899823).

## Additional Files

## Competing interests

The authors declare that they have no competing interests.

## Authors’ contributions

GZ analyzed data and drafted manuscript. EC performed the gel blots experiments. KKV, SB, and HW processed the Illumina small RNA raw sequence files and performed BLAST and BLAT searches to multiple databases. JT, SJ, BC and SC prepared tissues and RNA samples for sequencing. MH provided bioinformatics expertise. LOV obtained funding, coordinated and led the research, drafted sections, and edited the manuscript. All authors read and approved the final manuscript.

## Supplementary Material

Additional file 1 **Excel spreadsheet with 135,055 unique sequence signatures and their counts in each of the eight soybean tissue populations examined in the present study (WS, SCR, SCM, SCW, Cot, GCot ST, and LE). **See Table 1 for tissue descriptions and materials and methods for annotation descriptions. These files contain total non-normalized read counts. Click here for file

Additional file 2 **Excel file of unique small RNA sequence-reads bearing high similarity to annotated miRNAs obtained from the eight soybean tissue populations examined in the present study (WS, SCR, SCM, SCW, Cot, GCot ST, and LE).** See Table 1 for tissue descriptions and materials and methods for annotation descriptions. These files contain total non-normalized read counts. Click here for file

Additional file 3 Alignment of 218 tasiRNA unique signatures to the Glyma09g03730.1 genomic sequence. Click here for file

Additional file 4 Cis-acting regulatory motifs found on the promoter of the Glyma15g06080 gene encoding miR3522 as determined by PLACE, a database of plant cis-acting regulatory DNA elements (http://www.dna.affrc.go.jp/PLACE/signalscan.html) [59]. Click here for file
