# Supplementary material for: Divergent patterns of endogenous small RNA populations from seed and vegetative tissues of Glycine max
Source: BMC Plant Biol. 2012 Oct 2;12:177. doi: 10.1186/1471-2229-12-177 (PMC3534067; doi:10.1186/1471-2229-12-177)
Supplement: Additional file 3 — Alignment of 218 tasiRNA unique signatures to the Glyma09g03730.1 genomic sequence. [file 1471-2229-12-177-S3.doc]

**Figure S3.-** Glyma09g03730.1 putative ta-siRNA (*TAS3*)

🡪 ID#: 480674 Gma-miR390b


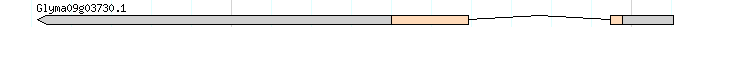


1 130

Glyma09g03 ATCTTACCAT AACAAAAAGT CCTCTCTCTC TCTCTCTCTT TCACACTCTC ACTTTTTCTG TGCTTCTCTT CTCTCTCCTT TCACCCTCTT GGAATAATAG AGTAGGGAGA AAAAAAAGGG TCTCAAAGAT

Exon1

131 260

Glyma09g03 GAGACCAAAA GACGGGTTTT TCATCAAAGG TACTATACTA ATACTAGTGT ACAAGTGTGT GTGTTGAGCT TGTAAAATAA AATTTGCATC ATATAGAATC ATAGCTTGCA CTGCACAGTT ATTTAGAACC

261 390

Glyma09g03 TTTTTAATAT GTGTAATTCA TATATGTTTG TACTAGCTTA TGCCATAAGA TAATTATTCT GTGACTGACT GAACTATTTA CTTTATGGTA ACTAAGATAT TCTCTTGTAT TCTTTTAATT GTTTTTATGT

391 520

Glyma09g03 TCACTTTTTT CTATCCTCCC CTTACTTTTT CATCTAATCT AAAGCTGGCT CTCCTGCTTC TGCTTCTGCC ATTCTTCTTC ATTATTCTCT GCACTAATTT TAAGAGCATG ATACATGATA CAGGTCCCTA

521 650

Glyma09g03 TAAGGGAGGG TTTTTTCTCA TGCATGTGGC TAGCAGCAGC AGCAAGTGCA AGGTGGCCAT GAGCATGACC ATGGTCAACG ACCCTTTGAG GAGAATTGGG AGGTGTGACC AAAAAAAAGA AGCCATTAGT

289287 TTTACTACT ACTACTACCC TTTCT

257979 TTACTACT ACTACTACCC TTTCTT

249760 TTTACTACT ACTACTACCC TTT

257978 CTACT ACTACTACCC TTTCTT

289160 CTACT ACTACTACCC TTTCTTTCT

371446 ACT ACTACTACCC TTTCTTTCTT C

289158 CT ACTACTACCC TTTCTTTCT

257943 T ACTACTACCC TTTCTTTCTT

442218 ACTACTACCC TTTCTTTCTT CA

442217 CTACTACCC TTTCTTTCTT CA

299096 CTACTACCC TTTCTTTCTT CATCT

299095 CTACCC TTTCTTTCTT CATCT

Exon2 415502 TACCC TTTCTTTCTT CATCTA

415503 AACCC TTTCTTTCTT CATCTA

468454 ACCC TTTCTTTCTT CATCTAA 780

461426 TTTCTT CATCTAATTT CTACCA

461425 TTCTT CATCTAATTT CTACCA (2,188 t.c.)

461431 TGCTT CATCTAATTT CTACCA

461432 TTCGT CATCTAATTT CTACCA

461430 ATCTT CATCTAATTT CTACCA

461429 GTCTT CATCTAATTT CTACCA

404089 TCTT CATCTAATTT CTACCAC

464933 CTT CATCTAATTT CTACCACA

461424 TCTT CATCTAATTT CTACCA

404486 TT CATCTAATTT CTACCACAC

266661 T CATCTAATTT CTACCACACT

315130 T CATCTAATTT CTACCACACT

418037 T CATCTAATTT CTACCACACT

418036 CATCTAATTT CTACCACACT

250213 ATCTAATTT CTACCACACT

376371 AATTT CTACCACACT

651 294831 ATTT CTACCACACT

Glyma09g03 GGCTCTGAAA TTGGAGCTGA CAATTCTCCT GCAGCAGGAG CAGCTTCTTC ATTCTAATCT GGTGCTATCC TATCTGAGCT TTTTACTACT ACTACTACCC TTTCTTTCTT CATCTAATTT CTACCACACT

345272 CATCTAATTT CTACCACACT

412421 TACCACACT

3’UTR-----🡪

337572 TTCCCTT GGAGTCTTCT TCTTG

258353 TTCCCTT GGAGTCTTCT TCTT

337571 TCCCTT GGAGTCTTCT TCTTG

422085 CCCTT GGAGTCTTCT TCTTGA

337576 TTTTCCCTT GGAGTCTTCT TG

422088 TTTCCCTT GGAGTCTTCT TGA

311760 TT GGAGTCTTCT TCTTGACCT

265154 TT GGAGTCTTCT TCTTGACCTT

265153 T GGAGTCTTCT TCTTGACCTT

338516 T GGAGTCTTCT TCTTGACCTT G

311762 AT GGAGTCTTCT TCTTGACCT

311761 GT GGAGTCTTCT TCTTGACCT

311759 T GGAGTCTTCT TCTTGACCT

265155 A GGAGTCTTCT TCTTGACCTT

338515 GGAGTCTTCT TCTTGACCTT G

274402 GGAGTCTTCT TCTTGACCTT GT TTCTACCACA CTTTTCTCTT T 248318

274401 GAGTCTTCT TCTTGACCTT GT TACCACA CTTTTCTCTT TGTT 252389

411773 AGTCTTCT TCTTGACCTT GTA ACCACA CTTTTCTCTT TGTTT 548381

439528 AGTCT TCTTGACCTT GTAAGA CACA CTTTTCTCTT TGTTTTT 243896

439527 CTTCT TCTTGACCTT GTAAGA T CACACCCTAT CTCTTCTCTT 259645

TTCTTTG TTTTTCCCCT GGAG 363960 CACACCCTAT CTCTTCTCTT T 248334

TTG TTTTTCCCTT GGAGTCTT 259335 CACACCCTAT CTCTTCTCTT TG 336121

337499 ACCTT GTAAGACCTT TTCTTG ACACCCTAT CTCTTCTCTT TG 336120

338513 TAAGACCTT TTCTTGACCT TG ACACCCTAT CTCTTCTCTT TA 407758

411772 AGACCTT TTCTTGACCT TGTA 243897 CTAT CTCTTCTCTT TGTTTTT 910

314745 AG ACCCTGTATC ACTATCCACT

314744 G ACCCTGTATC ACTATCCACT

424924 ACCCTGTATC ACTATCCACT

**Glyma09g03730.1** 🡨---**Glyma only**-🡪 424923 CCCTGTATC ACTATCCACT (2,087 t.c.)

344235 ACCCTGTATC ACTATCCACT

424927 CCCTATATC ACTATCCACT

424926 CCCGGTATC ACTATCCACT

323096 CCCTGTATC ACTATCCACT

781 323095 CCTGTATC ACTATCCACT

T 266661 418991 CTGTATC ACTATCCACT

315130 428402 CCCTGTATC ACTATCCACG

A 418037 417262 CT TGTAAGACCT CACACCCTA 434101 CCCTGTATC ACTATCCACC

A 418036 320535 T TGTAAGACCT CACACCCTAT 424922 CCTGTATC ACTATCCACT

TT 250213 295971 AAGACCT CACACCCTAT CTCT 314743 CCCTGTATC ACTATCCACT

TTTCTC 376371 259886 AGACCT CACACCCTAT CTCTT 344970 CTATATC ACTATCCACT

TTTCTCT 294831 290875 ACCT CACACCCTAT CTCTTCT 256945 C ACTATCCACT

Glyma09g03 TTTCTCTTTG TTTTTCCCTT GGAGTCTTCT TCTTGACCTT GTAAGACCTT TTCTTGACCT TGTAAGACCT CACACCCTAT CTCTTCTCTT TGTTTTTGCT TTTGTGGAAG ACCCTGTATC ACTATCCACT

T 345272 TTCTTGACCT TGTAAGACCT CA 474605

TTTCTCTTTG TT 412421 TCTTGACCT TGTAAGACCT CAC 440103

TG TTTTTCCCTT GGAGTCTTC 466204 TTCTTGACCT TGTAAGACCT C 474604 (10,565 t.c.)

TCTTGACCT TGTAAGACCT CA 440100 (20,866 t.c.)

TCTTGACCT TGTAAGACCT CAT 440104

TCTTGACCT TGTANGACCT CA 440164

TCGTGACCT TGTAAGACCT CA 434582

TCATGACCT TGTAAGACCT CA 425585

TCCTGACCT TGTAAGACCT CA 428851

TCTTGAACT TGTAAGACCT CA 440012

TCTTGACCT TGTCAGACCT CA 440173

TCTTGACCT TGTACGACCT CA 440155

TCTTGACCT TGTAAGCCCT CA 440136

TCTTGACCT TGTAAGACTT CA 440122

TCTTGACCT TGTAATACCT CA 440146

TCTTGACCT TGTAGGACCT CA 440163

TTCTTGACCT TGTAATACCT C 474625

TTCTTGACCT TGTAAGACAT C 474592

TTGTTGACCT TGTAAGACCT C 480845

TTATTGACCT TGTAAGACCT C 469769

TTCTTGACCT TGTAAGCCCT C 474620

TTCTTGACCT TGTACGACCT C 474628

TCTCGACCT TGTAAGACCT CA 436880

TTCTTGACCT TGTAAGAACT C 474587

TCTTGACCT TGTAAGACAT CA 440075

TCTTGACCT TGTAAGAACT CA 440069

TTCTTGACCT TGTAAGACCC C 474599

TTCTTGAACT TGTAAGACCT C 474553

TTCTTGACCT TGAAAGACCT C 474572

TCTTGACCT TGTAAGACCT GA 440111

TTCTTGACCT TGTAAGACTT C 474614

TTCTTGACCT TGTAAGATCT C 474616

TTCCTGACCT TGTAAGACCT C 471874

TTCTCGACCT TGTAAGACCT C 473602

TTCTTGACCT TGTAAAACCT C 474583

TCTTGACAT TGTAAGACCT CA 440037

TCTTGACCT TGTAAGACCT AA 440097

TTCTTGCCCT TGTAAGACCT C 474662

TCTTGACCT TGTAAAACCT CA 440058

TCTTGACCT TGTAAGACCT CC 440108

TTCTTGACCT TGTACGCCCT C 474630

TCTTGACCT TGTACGCCCT CA 440162

TTCTTGACCT TGTCAGCCCT C 474636

TCTTGACCT TGTAAGACCC C 440090

TCTTGACCT TGTACGACCT CC 440156

TCTTGACCT TGTAAGCCCT CC 440137

TTGACCT TGTAAGACCT CA 476313

TAAGACCTT TTCTTGACCT TG 409615

AAGACCTT TTCTTGACCT TGT 259085

439966 TCT TCTTGACCTT GTAAGACC

485824 TTT TCTTGACCTT GTAAGACC

368544 CT TCTTGACCTT GTAAGACCT

482970 TT TCTTGACCTT GTAAGACCT

474609 T TCTTGACCTT GTAAGACCTT (1,852 t.c.)

474602 T TCTTGACCTT GTAAGACCT

474626 T TCTTGACCTT GTAATACCTT

474603 T TCTTGACCTT GTAAGACCTA

474629 T TCTTGACCTT GTACGACCTT

474621 T TCTTGACCTT GTAAGCCCTT

474593 T TCTTGACCTT GTAAGACATT

440113 TCTTGACCTT GTAAGACCTT A

474615 T TCTTGACCTT GTAAGACTTT

440112 TCTTGACCTT GTAAGACCTT

440114 TCTTGACCTT GTAAGACCTT T

440116 TCTTGACCTT GTAAGACCTT TT

368664 CTTGACCTT GTAAGACCTT TT

368665 CTTGACCTT GTAAGACCTT TTC

440110 TCTTGACCTT GTAAGACCTC T

440099 TCTTGACCTT GTAAGACCTC

474594 T TCTTGACCTT GTAAGACC

440096 TCTTGACCTT GTAAGACCT

368663 CTTGACCTT GTAAGACCTT T

440098 TCTTGACCTT GTAAGACCTA C

440158 TCTTGACCTT GTACGACCTT T

440175 TCTTGACCTT GTCAGACCTT T

474596 T TCTTGACCTT GTAAGACCC

440138 TCTTGACCTT GTAAGCCCTT T

474634 T TCTTGACCTT GTCAGACCTC

440174 TCTTGACCTT GTCAGACCTC C

440123 TCTTGACCTT GTAAGACTTT

440078 TCTTGACCTT GTAAGACC

474589 T TCTTGACCTT GTAAGAC

476320 TTGACCTT GTAAGACCTT TTC

476321 TTGACCTT GTAAGACCTT TTCT

445976 TGACCTT GTAAGACCTT TTCT (5,580 t.c.)

445978 TGACCTT GTAAGACCTT TTCTT

445986 TGACCTT GTCAGACCTT TTCT

445960 TGACCTT GTAAGACCCT TTCT

476322 TTGACCTT GTAAGACCTT TTT

445972 TGACCTT GTAAGACCTT TTAT

445980 TGACCTT GTAAGACCTT TTT

445981 TGACCTT GTAAGACCTT TTTT

445957 TGACCTT GTAAGAACTT TTCT

445983 TGACCTT GTAAGCCCTT TTCT

459562 TGGCCTT GTAAGACCTT TTCT

445973 TGACCTT GTAAGACCTT TTC

445975 TGACCTT GTAAGACCTT TTCC

476310 TTGACCTT GTAAGACATT TTC

445958 TGACCTT GTAAGACATT TTCT

476318 TTGACCTT GTAAGACCTT TT

476319 TTGACCTT GTAAGACCTT TTA

445971 TGACCTT GTAAGACCTT TT

476317 TTGACCTT GTAAGACCTT T

445969 TGACCTT GTAAGACCTT T

440142 TCTTGACCTT GTAATACC

440166 TCTTGACCTT GTCAGACC

474585 T TCTTGACCTT GTAAGA

476311 TTGACCTT GTAAGACC

440131 TCTTGACCTT GTAAGCCC

440148 TCTTGACCTT GTACGACC

440071 TCTTGACCTT GTAAGAC

440072 TCTTGACCTT GTAAGACA

352768 CCTT GTAAGACCTT TTCTTGA

445959 TGACCTT GTAAGACC

440046 TCTTGACCT CGTAAGACCT CA 455962 TGCT TTTGTGGAAG ACCCTGTATC

440204 TCTTGACTT TGTAAGACCT CA 478205 TTGCT TTTGTGGAAG ACCCTGT

440294 TCTTGCCCT TGTAAGACCT CA 455960 TGCT TTTGTGGAAG ACCCTGTA

440187 TCTTGACCT TGTGAGACCT CA 483944 TTTGCT TTTGTGGAAG ACCCTG

440080 TCTTGACCT TGTAAGACCA CA 478204 TTGCT TTTGTGGAAG ACCCTG

440111 TCTTGACCT TGTAAGACCT GA 455959 TGCT TTTGTGGAAG ACCCTGT

440128 TCTTGACCT TGTAAGATCT CA 389680 GCT TTTGTGGAAG ACCCTGTA

440060 TCTTGACCT TGTAACACCT CA 389681 GCT TTTGTGGAAG ACCCTGTAT

439468 TCTTAACCT TGTAAGACCT CA 486536 TTTTTGCT TTTGTGGAAG ACC

440599 TCTTTACCT TGTAAGACCT CA 486537 TTTTTGCT TTTGTGGAAG ACCC

546540 TCTTCACCT TGTAAGACCT CA 486025 TTTTGCT TTTGTGGAAG ACCC

440048 TCTTGACCT TATAAGACCT CA 483943 TTTGCT TTTGTGGAAG ACCCT

440070 TCTTGACCT TGTAAGAACT CC 483946 TTTGCT TTTGTGGAAG ACCCTT

440076 TCTTGACCT TGTAAGACAT CC 478206 TTGCT TTTGTGGAAG ACCCTT

440179 TCTTGACCT TGTCAGCCCT CA 478203 TTGCT TTTGTGGAAG ACCCT

440185 TCTTGACCT TGTCCGACCT CA 478201 TTGCT TTTGTGGAAG AACCTG

440068 TCTTGACCT TGTAAGAACT AA 455957 TGCT TTTGTGGAAG ACCCTG

440074 TCTTGACCT TGTAAGACAT AA 455964 TGCT TTTGTGGAAG ACCCTTT

368662 CTTGACCT TGTAAGACCT CAC 455952 TGCT TTTGTGGAAG AACCTGT

476314 TTGACCT TGTAAGACCT CACA 455965 TGCT TTTGTGGAAG CCCCTGT

445964 TGACCT TGTAAGACCT CACAC 455966 TGCT TTTGTGGACG ACCCTGT

445965 TGACCT TGTAAGACCT CACACC 455953 TGCT TTTGTGGAAG ACACTGT

445967 TGACCT TGTAAGACCT CACCC 483942 TTTGCT TTTGTGGAAG ACCC

445962 TGACCT TGTAAGACCT CACA 369717 CT TTTGTGGAAG ACCCTGTT

445968 TGACCT TGTAAGACCT CCCAC 369718 CT TTTGTGGAAG ACCCTGTTT

476313 TTGACCT TGTAAGACCT CA 369712 CT TTTGTGGAAG ACCCTGTA

440123 TCTTGACCT TGTAAGACTT T 486539 TTTTTGCT TTTGTGGACG ACC

368663 CTTGACCT TGTAAGACCT TT 486535 TTTTTGCT TTTGTGGAAG ACA

476317 TTGACCT TGTAAGACCT TT 486534 TTTTTGCT TTTGTGGAAG AC

440072 TCTTGACCT TGTAAGACA 486538 TTTTTGCT TTTGTGGAAG CCC

440166 TCTTGACCT TGTCAGACC 486533 TTTTTGCT TTTGTGGAAG AAC

281298 ACCT TGTAAGACCT CACACCCT 486542 TTTTTGCT TTTGTTGAAG ACC

281297 ACCT TGTAAGACCT CACACCC 486541 TTTTTGCT TTTGTGTAAG ACC

352766 CCT TGTAAGACCT CACACCCT 600136 GCT TTTGTGGAAG ACCCTGT

352767 CCT TGTAAGACCT CACACCCTA 483940 TTTGCT TTTGTGGAAG ACC

369106 CT TGTAAGACCT CACACCCTA 369713 CT TTTGTGGAAG ACCCTGTAT

369107 CT TGTAAGACCT CACACCCTAT 369714 CT TTTGTGGAAG ACCCTGTATC

479902 T TGTAAGACCT CACACCCTAT 486244 T TTTGTGGAAG ACCCTGTATC

479903 T TGTAAGACCT CACACCCTAT C 486245 T TTTGTGGAAG ACCCTGTATC A

445969 TGACCT TGTAAGACCT TT 486249 T TTTGTGGAAG ACCCTGTCTC

476311 TTGACCT TGTAAGACC 486243 T TTTGTGGAAG ACCCTGTATA

445959 TGACCT TGTAAGACC 485038 TTTGTGGAAG ACCCTGTATC C

485040 TTTGTGGAAG ACCCTGTATC T

485034 TTTGTGGAAG ACCCTGTATC

369710 CT TTTGTGGAAG AACCTGTAT

485043 TTTGTGGAAG ACCCTGTTTC A

486241 T TTTGTGGAAG ACCCTGTA

485036 TTTGTGGAAG ACCCTGTATC AC

485035 TTTGTGGAAG ACCCTGTATC A

480557 TTGTGGAAG ACCCTGTATC AC

480558 TTGTGGAAG ACCCTGTATC ACT

480559 TTGTGGAAG ACCCTGTATC ACTT

403468 GTGGAAG ACCCTGTATC ACTT

480555 TTGTGGAAG ACCCTGTATC A

403467 GTGGAAG ACCCTGTATC ACTA

480561 TTGTGGAAG ACCCTGTATT T

456456 TGGAAG ACCCTGTATC ACTAT

349548 CCCTGTATC ACTATCCACT

363200 CTATCCACT

419076 TATCCACT

426580 TCCACT

302409 TCTTCT TTCCCCGCT

451324 TCCCCG TTACCACCCA ACTCA (1,108 t.c.)

451326 CCCCCG TTACCACCCA ACTCA

451327 ACCCCG TTACCACCCA ACTCA

451325 GCCCCG TTACCACCCA ACTCA

455268 TCCCCG TTACCACCCA ACGCA

328075 CCCCG TTACCACCCA ACTCAT

451323 CCCCG TTACCACCCA ACTCA

419802 CCCG TTACCACCCA ACTCATA

401331 CCG TTACCACCCA ACTCATAC

249992 TTACCACCCA ACTCATACTT T

383860 ATGTA GAGTTTGATC TCCTGC

261324 TGTA GAGTTTGATC TCCTGCTT

265818 TGTA GAGTTTGATC TCCTACTT

261323 GTA GAGTTTGATC TCCTGCTT

265817 GTA GAGTTTGATC TCCTACTT

258579 TATA GAGTTTGATC TCCTTCTT

258578 ATA GAGTTTGATC TCCTTCTT

248901 TA GAGTTTGATC TCCTGCTTT

249959 TA GAGTTTGATC TCCTACTTT

359657 TTTGATC TCCTTCTTTC CCCG

283760 TTGATC TCCTTCTTTC CCCGT

283761 TTGATC TCCTACTTTC CCCGT

256102 TGATC TCCTTCTTTC CCCGTT

256103 TGATC TCCTACTTTC CCCGTT

408802 GATC TCCTTCTTTC CCCGTTA

399792 ATC TCCTTCTTTC CCCGTTAC

461284 C TCCTTCTTTC CCCGTTACCA

404081 TCCTTCTTTC CCCGTTACCA C

303861 CCACCCA ACTCATACTT TCCT

303860 CACCCA ACTCATACTT TCCT

303863 AAACCCA ACTCATACTT TCCT

262734 CACCCA ACTCATACTT TCCTT

372481 ACCCA ACTCATACTT TCCTTC

262733 ACCCA ACTCATACTT TCCTT

262735 AACCCA ACTCATACTT TCCTT

303862 AACCCA ACTCATACTT TCCT

304305 CCA ACTCATACTT TCCTTCCT

262963 CA ACTCATACTT TCCTTCCTT

338208 CA ACTCATACTT TCCTTCCTTG

338207 A ACTCATACTT TCCTTCCTTG

414761 CATACTT TCCTTCCTTG TCTA

414760 ATACTT TCCTTCCTTG TCTA

319761 ATACTT TCCTTCCTTG TCTAT

380206 ATACTT TCCTTCCTTG TCTATC

380205 TACTT TCCTTCCTTG TCTATC

319760 TACTT TCCTTCCTTG TCTAT

911 414759 TACTT TCCTTCCTTG TCTA

CT 290875 309674 T TCCTTCCTTG TCTATCCCT

314745 378850 T TCCTTCCTTG TCTATCCCTC

314744 TTCCTTG TCTATCCCTC CTGA 424526

GA 424924 TCCTTG TCTATCCCTC CTGAG 363747

GA 424923 TTG TCTATCCCTC CTGAGCTG 342974

G 344235 G TCTATCCCTC CTGAGCTGTT 253533

GA 424927 TATCCCTC CTGAGCTGTT CCA 456310

GA 424926 TATCCCTC CTGAGCTGTT CCAA 478271

GAT 323096 ATCCCTC CTGAGCTGTT CCAA 478270

GAT 323095 ATCCCTC CTGAGCTGTT CCAG 366481

GATA 418991 TCCCTC CTGAGCTGTT CCAAT 333797

GA 428402 CCCTC CTGAGCTGTT CCAATT 272551

GA 434101 CCCTC CTGAGCTGTT CCAGTT 257224

GA 424922 CTC CTGAGCTGTT CCAATTTA 408313

314743 CTC CTGAGCTGTT CCAGTTTA 407645

GATG 344970 CTC CTGAGCTGTT CCAGTTTAA 466371

GATATAGAGT T 256945 TC CTGAGCTGTT CCAGTTTAA 466370 1040

Glyma09g03 GATATAGAGT TTGATCTCCT TCTTTCCCCG TTACCACCCA ACTCATACTT TCCTTCCTTG TCTATCCCTC CTGAGCTGTT CCAATTTAAT TAATTTGGCC TACCATATAT GATGCAACAA TTTAATGTAA

349548 GA CCACCCA ACTCATACTT TCCT 347359 553603 TGGCC TACCATATAT GATGCA

363200 GATATAGAGT TTG CACCCA ACTCATACTT TCCTT 340300

419076 GATATAGAGT TTGA ACCCA ACTCATACTT TCCTTC 279665

426580 GATATAGAGT TTGAT CCCA ACTCATACTT TCCTTCC 348478

321148 ATATAGAGT TTGATCTCCT TC CTT TCCTTCCTTG TCTATCCC 369366

551502 TAGAGT TTGATCTCCT TCTTTC

GGTCG TCTATCCCTC CTGAGCTT  **miR390b**

GG-CG -CTATCCCTC CTGAGCTT **miR390a**

1170

416027 TGCC

465845 GGCTTCC

1041 416027 TGCCAAATGT GTTGTTAGCT A 339665 TCC

Glyma09g03 TATTATGCCA TACCATGGTA TCATATGGTA TGGTATAGCT TCATAGAAAT TAATTAAGGA CCTTACCTTA ACCTTAGAAG TTACAACCTC TGCCAAATGT GTTGTTAGCT ATTTGGAGAC CATGGCTTCC

425226 TCATATGGTA TGGTATAGCT T

321849 ATATGGTA TGGTATAGCT TCA

420293 TATGGTA TGGTATAGCT TCAT

461615 TGGTA TGGTATAGCT TCATAG

1171

AAGAGAGTAC AACA 465845

AAGAGAGTAC AACAATTG 339665

GAGAGTAC AACAATTGGG GTA 412516 243605 TTTT TTTTTTTTTT T 328247 ATGTAGT TCGTGCAGCA TCAT 1300

Glyma09g03 AAGAGAGTAC AACAATTGGG GTATATTATT TTTATTTTAT TTGATATTTT TTTTTTATCT TGGTGAGAAA TTAATGTAGT TCGTGCAGCA TCATGGTTGG TCGTGGTCCT TAAGGTATCT GTGGTAAGTT

251717 AAA TTAATGTAGT TCGTGCAG TTGG TCGTGGTCCT TAAGGTA 479663

250937 AAA TCAAAGTAGT TCGTGCAG TTGG TCGTGGTCCT TAAGGTAT 479664

467316 TTAATGTAGT TCGTGCAGCA T TGG TCGTGGTCCT TAAGGTAT 461798

467317 TTAATGTAGT TCGTGCAGCA TC

411299 TAATGTAGT TCGTGCAGCA TC

330811 ATGTAGT TCGTGCAGCA TCAT

1301 471853 TGCTGCTA GTATTCAGAG GAA 1430

Glyma09g03 GTAAAGAGTG GCCATAGTTA ATAATAAATC AATGCTGCTA GTATTCAGAG GAAATTCAAA AGCCAAATCT GTATCATATA TATTTCTGTT ATGGCAATCA ATTTCGCTTG TATCATTGTG TGCAGAATAT

1431 1560

Glyma09g03 TTATGCTGTG TTGCAGACAT CTGTTTCGTT GTTTTATATG TTGTTTGGCT GAGAAAGTGT GCAACAACTG CATACCATAT TATTCTTTTA GATCTAAGCA AGAGTCTCCC TCGTGACTGC TCCAATGTAC

1561 1590

Glyma09g03 AAATATCCAC TGTATTATTG GAGTTATCTC
