# Supplementary material for: Divergent patterns of endogenous small RNA populations from seed and vegetative tissues of Glycine max
Source: BMC Plant Biol. 2012 Oct 2;12:177. doi: 10.1186/1471-2229-12-177 (PMC3534067; doi:10.1186/1471-2229-12-177)
Supplement: Additional file 4 — Cis-acting regulatory motifs found on the promoter of the Glyma15g06080 gene encoding miR3522 as determined by PLACE, a database of plant cis-acting regulatory DNA elements (http://www.dna.affrc.go.jp/PLACE/signalscan.html) [59]. [file 1471-2229-12-177-S4.doc]

**Glyma15g06080.1**

**miR3522: TGAGACCAAATGAGCAGCTGA**

**>Gm15:4318065..4320064**

**CTTTATGTAAGCATATGATTTCATTTCATCATTGCTTAGCACTTTTTGtatttttaaaaa**

**attctcaaatcattaattattttaaattttgtatttttttttaaatCTCAGTTACTTGCA**

**CTAAAATAATTATATGTTGGTCCCTTTTTACATTAAATTTCTTTTTCTTTCCCCAAACTT**

**TAACTACTTAAATTCTCaaattaattatttttaataaaaaattaCTTTCTCAAATCTAAG**

**CTGATCCACTCTTACATTAAGAAGACACGCTATTAACTTTTCTTTTATCAAATTAATTAT**

**ACATGTCTCtttataagttttttttttcttttgatatatatttattaaattCTACCTGAA**

**TCAATTATGtatataatgtttataattttaaatatttttttttttctttctaggttatat**

**ttttttaatttaaaatattGACGAAGTCAACAATATTTGTGTTTTGCATTGACGTGCTGG**

**CAACGGGAAGTTGTACTATATACATAGCGTCTTAGATTTGTGTTCAAATCAGTAGTACAA**

**AGTACTAGTACTACATGTACATCTAACCTAGAAGAAGTACCACGCTTGCTTGAGGCTATA**

**TATCCACTGAATCAAGTTGGATAAACATAAACTCATTACATTGATAAAACACAATTCAAA**

**AGATCAATGTTCCACTTCATGCAAAGACATTTCCAAAATATGTGTAGGTAGAGGGGTTTT**

**ACAGGATCGTCCTGAGACCAAATGAGCAGCTGACCACATGATGCAGCTATGTTTGCTATT**

**CAGCTGCTCATCTGTTCTCAGGTCGCCCTTGTTGGACTGTCCAACTCCTACTGATTGCGG**

**ATGCACTTGCCACAAATGAAAATCAAAGCGAGGGGAAAAGAATGTAGAGTGTGACTACGA**

**TTGCATGCATGTGATTTAGGTAATTAAGTTACATGATTGTCTAATTGTGTTTATGGAATT**

**GTATATTTTCAGACCAGGCACCTGTAACTAATTATAGGTACCATACCTTAAAATAAGTCC**

**AACTAAGTCCATGTCTGTGATTTTTTAGTGTCACAAATCACAATCCATTGCCATTGGTTT**

**TTTAATTTTTCATTGTCTGTTGTTTAACTAACTCTAGCTTTTTAGCTGCTTCAAGTACAG**

**ATTCCTCAAAGTGGAAAATGTTCTTTGAAGTCAATAAAAAGAGCTTTGATGATCATCTGC**

**ATTGTCTAAGTTGGATAAACTAATTAGAGAGAACTTTTGAACTTTGTCTACCAAATATCT**

**GTCAGTGTCATCTGTCAGTTCTGCAAGCTGAAGTGTTGAATCCACGAGGTGCTTGTTGCA**

**AAGTTGTGATATTAAAAGACATCTACGAAGAAGTTCAAGCAAAACTCTTTTTGGCATACT**

**RESULTS OF YOUR SIGNAL SCAN SEARCH REQUEST**

../../tmp/sigscan//signalseqdone.12083: 1375 base pairs

Signal database file: user.dat

(+) = Current Strand

1 CTTTATGTAAGCATATGATTTCATTTCATCATTGCTTAGCACTTTTTGta

(+)CATATGGMSAUR [S000370](http://www.dna.affrc.go.jp/sigscan/disp.cgi?S000370)

(+)EBOXBNNAPA [S000144](http://www.dna.affrc.go.jp/sigscan/disp.cgi?S000144)

(+)MYCCONSENSUSAT [S000407](http://www.dna.affrc.go.jp/sigscan/disp.cgi?S000407)

(+)ARR1AT [S000454](http://www.dna.affrc.go.jp/sigscan/disp.cgi?S000454)

(+)INRNTPSADB [S000395](http://www.dna.affrc.go.jp/sigscan/disp.cgi?S000395)

(+)CACTFTPPCA1 [S000449](http://www.dna.affrc.go.jp/sigscan/disp.cgi?S000449)

(+)SEF4MOTIFGM7S [S000103](http://www.dna.affrc.go.jp/sigscan/disp.cgi?S000103)

51 TTTTTAAAAAATTCTCAAATCATTAA**TTATTT**TAAATTTTGTATTTTTTT

(+)TATABOX5 [S000203](http://www.dna.affrc.go.jp/sigscan/disp.cgi?S000203)

101 TTAAATCTCAGTTACTTGCACTAAAATAATTATATGTTGGTCCCTTTTTA

(+)MYBCORE [S000176](http://www.dna.affrc.go.jp/sigscan/disp.cgi?S000176)

(+)CACTFTPPCA1 [S000449](http://www.dna.affrc.go.jp/sigscan/disp.cgi?S000449)

(+)CACTFTPPCA1 [S000449](http://www.dna.affrc.go.jp/sigscan/disp.cgi?S000449)

(+)POLASIG3 [S000088](http://www.dna.affrc.go.jp/sigscan/disp.cgi?S000088)

(+)PYRIMIDINEBOXOSRAMY1A [S000259](http://www.dna.affrc.go.jp/sigscan/disp.cgi?S000259)

151 CATTAAATTTCTTTTTCTTTCCCCAAACTTTAACTACTTAAATTCTCaaa

(+)NTBBF1ARROLB [S000273](http://www.dna.affrc.go.jp/sigscan/disp.cgi?S000273)

(+)CACTFTPPCA1 [S000449](http://www.dna.affrc.go.jp/sigscan/disp.cgi?S000449)

201 TTAA**TTATTT**TTAATAAAAAATTACTTTCTCAAATCTAAGCTGATCCACT

(+)TATABOX5 [S000203](http://www.dna.affrc.go.jp/sigscan/disp.cgi?S000203)

(+)SEF4MOTIFGM7S [S000103](http://www.dna.affrc.go.jp/sigscan/disp.cgi?S000103)

(+)POLASIG1 [S000080](http://www.dna.affrc.go.jp/sigscan/disp.cgi?S000080)

(+)CACTFTPPCA1 [S000449](http://www.dna.affrc.go.jp/sigscan/disp.cgi?S000449)

(+)CACTFTPPCA1 [S000449](http://www.dna.affrc.go.jp/sigscan/disp.cgi?S000449)

(+)NODCON2GM [S000462](http://www.dna.affrc.go.jp/sigscan/disp.cgi?S000462)

(+)OSE2ROOTNODULE [S000468](http://www.dna.affrc.go.jp/sigscan/disp.cgi?S000468)

251 CTTACATTAAGAAGACACGCTATTAACTTTTCTTTTATCAAATTAATTAT

301 ACATGTCTCtttataagttttttttttcttttgatatatatttattaaat

(+)ARFAT [S000270](http://www.dna.affrc.go.jp/sigscan/disp.cgi?S000270)

351 TCTACCTGAATCAATTATG**TATATAA**TGTTTATAATTTTAAATATTTTTT

(+)CAATBOX1 [S000028](http://www.dna.affrc.go.jp/sigscan/disp.cgi?S000028)

(+)TATABOX4 [S000111](http://www.dna.affrc.go.jp/sigscan/disp.cgi?S000111)

(+)ROOTMOTIFTAPOX1 [S000098](http://www.dna.affrc.go.jp/sigscan/disp.cgi?S000098)

(+)MARTBOX [S000067](http://www.dna.affrc.go.jp/sigscan/disp.cgi?S000067)

(+)MARTBOX [S000067](http://www.dna.affrc.go.jp/sigscan/disp.cgi?S000067)

401 TTTTTCTTTCTAGGTTATATTTTTTTAATTTAAAATATTGACGAAGTCAA

(+)MARTBOX [S000067](http://www.dna.affrc.go.jp/sigscan/disp.cgi?S000067)

(+)ROOTMOTIFTAPOX1 [S000098](http://www.dna.affrc.go.jp/sigscan/disp.cgi?S000098)

(+)LECPLEACS2 [S000465](http://www.dna.affrc.go.jp/sigscan/disp.cgi?S000465)

(+)ROOTMOTIFTAPOX1 [S000098](http://www.dna.affrc.go.jp/sigscan/disp.cgi?S000098)

(+)WBOXATNPR1 [S000390](http://www.dna.affrc.go.jp/sigscan/disp.cgi?S000390)

(+)ASF1MOTIFCAMV [S000024](http://www.dna.affrc.go.jp/sigscan/disp.cgi?S000024)

(+)WRKY71OS [S000447](http://www.dna.affrc.go.jp/sigscan/disp.cgi?S000447)

(+)RAV1AAT [S000314](http://www.dna.affrc.go.jp/sigscan/disp.cgi?S000314)

451 CAATATTTGTGTTTTGCATTGACGTGCTGGCAACGGGAAGTTGTACTATA

(+)CAATBOX1 [S000028](http://www.dna.affrc.go.jp/sigscan/disp.cgi?S000028) TACT

(+)ROOTMOTIFTAPOX1 [S000098](http://www.dna.affrc.go.jp/sigscan/disp.cgi?S000098)

(+)WBOXATNPR1 [S000390](http://www.dna.affrc.go.jp/sigscan/disp.cgi?S000390)

(+)ASF1MOTIFCAMV [S000024](http://www.dna.affrc.go.jp/sigscan/disp.cgi?S000024)

(+)TGACGTVMAMY [S000377](http://www.dna.affrc.go.jp/sigscan/disp.cgi?S000377)

(+)WRKY71OS [S000447](http://www.dna.affrc.go.jp/sigscan/disp.cgi?S000447)

(+)ABRELATERD1 [S000414](http://www.dna.affrc.go.jp/sigscan/disp.cgi?S000414)

(+)ACGTATERD1 [S000415](http://www.dna.affrc.go.jp/sigscan/disp.cgi?S000415)

(+)MYB2CONSENSUSAT [S000409](http://www.dna.affrc.go.jp/sigscan/disp.cgi?S000409)

(+)MYBCOREATCYCB1 [S000502](http://www.dna.affrc.go.jp/sigscan/disp.cgi?S000502)

(+)CURECORECR [S000493](http://www.dna.affrc.go.jp/sigscan/disp.cgi?S000493)

(+)CACTFTPPCA1 [S000449](http://www.dna.affrc.go.jp/sigscan/disp.cgi?S000449)

501 TACATAGCGTCTTAGATTTGTGTTCAAATCAGTAGTACAAAGTACTAGTA

(+)ARR1AT [S000454](http://www.dna.affrc.go.jp/sigscan/disp.cgi?S000454) TACT TACT

(+)CURECORECR [S000493](http://www.dna.affrc.go.jp/sigscan/disp.cgi?S000493)

(+)DOFCOREZM [S000265](http://www.dna.affrc.go.jp/sigscan/disp.cgi?S000265)

(+)CURECORECR [S000493](http://www.dna.affrc.go.jp/sigscan/disp.cgi?S000493)

(+)CACTFTPPCA1 [S000449](http://www.dna.affrc.go.jp/sigscan/disp.cgi?S000449)

(+)CURECORECR [S000493](http://www.dna.affrc.go.jp/sigscan/disp.cgi?S000493)

(+)CACTFTPPCA1 [S000449](http://www.dna.affrc.go.jp/sigscan/disp.cgi?S000449)

551 CTACATGTACATCTAACCTAGAAGAAGTACCACGCTTGCTTGAGGCTATA

(+)CURECORECR [S000493](http://www.dna.affrc.go.jp/sigscan/disp.cgi?S000493)

(+)CURECORECR [S000493](http://www.dna.affrc.go.jp/sigscan/disp.cgi?S000493)

**TATCCAC**

601 TATCCACTGAATCAAGTTGGATAAACATAAACTCATTACATTGATAAAAC

(+)TATCCACHVAL21 [S000416](http://www.dna.affrc.go.jp/sigscan/disp.cgi?S000416)

(+)TATCCAOSAMY [S000403](http://www.dna.affrc.go.jp/sigscan/disp.cgi?S000403)

(+)TATCCAYMOTIFOSRAMY3D [S000256](http://www.dna.affrc.go.jp/sigscan/disp.cgi?S000256)

(+)CACTFTPPCA1 [S000449](http://www.dna.affrc.go.jp/sigscan/disp.cgi?S000449)

(+)MYBST1 [S000180](http://www.dna.affrc.go.jp/sigscan/disp.cgi?S000180)

(+)GATABOX [S000039](http://www.dna.affrc.go.jp/sigscan/disp.cgi?S000039)

(+)GT1CONSENSUS [S000198](http://www.dna.affrc.go.jp/sigscan/disp.cgi?S000198)

(+)IBOXCORE [S000199](http://www.dna.affrc.go.jp/sigscan/disp.cgi?S000199)

(+)PREATPRODH [S000450](http://www.dna.affrc.go.jp/sigscan/disp.cgi?S000450)

(+)GATABOX [S000039](http://www.dna.affrc.go.jp/sigscan/disp.cgi?S000039)

(+)GT1CONSENSUS [S000198](http://www.dna.affrc.go.jp/sigscan/disp.cgi?S000198)

(+)IBOXCORE [S000199](http://www.dna.affrc.go.jp/sigscan/disp.cgi?S000199)

651 ACAATTCAAAAGATCAATGTTCCACTTCATGCAAAGACATTTCCAAAATA

(+)CAATBOX1 [S000028](http://www.dna.affrc.go.jp/sigscan/disp.cgi?S000028)

(+)ERELEE4 [S000037](http://www.dna.affrc.go.jp/sigscan/disp.cgi?S000037)

(+)DOFCOREZM [S000265](http://www.dna.affrc.go.jp/sigscan/disp.cgi?S000265)

(+)NODCON1GM [S000461](http://www.dna.affrc.go.jp/sigscan/disp.cgi?S000461)

(+)OSE1ROOTNODULE [S000467](http://www.dna.affrc.go.jp/sigscan/disp.cgi?S000467)

(+)CAATBOX1 [S000028](http://www.dna.affrc.go.jp/sigscan/disp.cgi?S000028)

(+)CACTFTPPCA1 [S000449](http://www.dna.affrc.go.jp/sigscan/disp.cgi?S000449)

(+)RYREPEATBNNAPA [S000264](http://www.dna.affrc.go.jp/sigscan/disp.cgi?S000264)

(+)PROLAMINBOXOSGLUB1 [S000354](http://www.dna.affrc.go.jp/sigscan/disp.cgi?S000354)

(+)DOFCOREZM [S000265](http://www.dna.affrc.go.jp/sigscan/disp.cgi?S000265)

701 TGTGTAGGTAGAGGGGTTTTACAGGATCGTCCTGAGACCAAATGAGCAGC

(+)SURECOREATSULTR11 [S000499](http://www.dna.affrc.go.jp/sigscan/disp.cgi?S000499)

(+)EBOXBNNAPA [S000144](http://www.dna.affrc.go.jp/sigscan/disp.cgi?S000144)

(+)MYCCONSENSUSAT [S000407](http://www.dna.affrc.go.jp/sigscan/disp.cgi?S000407)

(+)ANAERO2CONSENSUS [S000478](http://www.dna.affrc.go.jp/sigscan/disp.cgi?S000478)

(+)EBOXBNNAPA [S000144](http://www.dna.affrc.go.jp/sigscan/disp.cgi?S000144)

(+)MYCCONSENSUSAT [S000407](http://www.dna.affrc.go.jp/sigscan/disp.cgi?S000407)

(-)EBOXBNNAPA [S000144](http://www.dna.affrc.go.jp/sigscan/disp.cgi?S000144)

(+)WBOXNTCHN48 [S000508](http://www.dna.affrc.go.jp/sigscan/disp.cgi?S000508)

RESULTS OF YOUR SIGNAL SCAN SEARCH REQUEST

../../tmp/sigscan//signalseqdone.12261: 1375 base pairs

Signal Database File: user.dat

Factor or Site Name Loc.(Str.) Signal Sequence SITE #

_____________________________________________________________________________________

ABRELATERD1 site 472 (+) ACGTG [S000414](http://www.dna.affrc.go.jp/sigscan/disp.cgi?S000414)

ACGTATERD1 site 472 (+) ACGT [S000415](http://www.dna.affrc.go.jp/sigscan/disp.cgi?S000415)

ANAERO2CONSENSUS site 745 (+) AGCAGC [S000478](http://www.dna.affrc.go.jp/sigscan/disp.cgi?S000478)

ARFAT site 304 (+) TGTCTC [S000270](http://www.dna.affrc.go.jp/sigscan/disp.cgi?S000270)

ARR1AT site 514 (+) NGATT [S000454](http://www.dna.affrc.go.jp/sigscan/disp.cgi?S000454)

ARR1AT site 16 (+) NGATT [S000454](http://www.dna.affrc.go.jp/sigscan/disp.cgi?S000454)

ASF1MOTIFCAMV site 439 (+) TGACG [S000024](http://www.dna.affrc.go.jp/sigscan/disp.cgi?S000024)

ASF1MOTIFCAMV site 470 (+) TGACG [S000024](http://www.dna.affrc.go.jp/sigscan/disp.cgi?S000024)

CAATBOX1 site 362 (+) CAAT [S000028](http://www.dna.affrc.go.jp/sigscan/disp.cgi?S000028)

CAATBOX1 site 451 (+) CAAT [S000028](http://www.dna.affrc.go.jp/sigscan/disp.cgi?S000028)

CAATBOX1 site 652 (+) CAAT [S000028](http://www.dna.affrc.go.jp/sigscan/disp.cgi?S000028)

CAATBOX1 site 665 (+) CAAT [S000028](http://www.dna.affrc.go.jp/sigscan/disp.cgi?S000028)

CACTFTPPCA1 site 40 (+) YACT [S000449](http://www.dna.affrc.go.jp/sigscan/disp.cgi?S000449)

CACTFTPPCA1 site 119 (+) YACT [S000449](http://www.dna.affrc.go.jp/sigscan/disp.cgi?S000449)

CACTFTPPCA1 site 247 (+) YACT [S000449](http://www.dna.affrc.go.jp/sigscan/disp.cgi?S000449)

CACTFTPPCA1 site 605 (+) YACT [S000449](http://www.dna.affrc.go.jp/sigscan/disp.cgi?S000449)

CACTFTPPCA1 site 673 (+) YACT [S000449](http://www.dna.affrc.go.jp/sigscan/disp.cgi?S000449)

CACTFTPPCA1 site 113 (+) YACT [S000449](http://www.dna.affrc.go.jp/sigscan/disp.cgi?S000449)

CACTFTPPCA1 site 185 (+) YACT [S000449](http://www.dna.affrc.go.jp/sigscan/disp.cgi?S000449)

CACTFTPPCA1 site 223 (+) YACT [S000449](http://www.dna.affrc.go.jp/sigscan/disp.cgi?S000449)

CACTFTPPCA1 site 494 (+) YACT [S000449](http://www.dna.affrc.go.jp/sigscan/disp.cgi?S000449)

CACTFTPPCA1 site 543 (+) YACT [S000449](http://www.dna.affrc.go.jp/sigscan/disp.cgi?S000449)

CACTFTPPCA1 site 549 (+) YACT [S000449](http://www.dna.affrc.go.jp/sigscan/disp.cgi?S000449)

CACTFTPPCA1 site 829 (+) YACT [S000449](http://www.dna.affrc.go.jp/sigscan/disp.cgi?S000449)

CATATGGMSAUR site 12 (+) CATATG [S000370](http://www.dna.affrc.go.jp/sigscan/disp.cgi?S000370)

CURECORECR site 493 (+) GTAC [S000493](http://www.dna.affrc.go.jp/sigscan/disp.cgi?S000493)

CURECORECR site 535 (+) GTAC [S000493](http://www.dna.affrc.go.jp/sigscan/disp.cgi?S000493)

CURECORECR site 542 (+) GTAC [S000493](http://www.dna.affrc.go.jp/sigscan/disp.cgi?S000493)

CURECORECR site 548 (+) GTAC [S000493](http://www.dna.affrc.go.jp/sigscan/disp.cgi?S000493)

CURECORECR site 557 (+) GTAC [S000493](http://www.dna.affrc.go.jp/sigscan/disp.cgi?S000493)

CURECORECR site 577 (+) GTAC [S000493](http://www.dna.affrc.go.jp/sigscan/disp.cgi?S000493)

DOFCOREZM site 539 (+) AAAG [S000265](http://www.dna.affrc.go.jp/sigscan/disp.cgi?S000265)

DOFCOREZM site 659 (+) AAAG [S000265](http://www.dna.affrc.go.jp/sigscan/disp.cgi?S000265)

DOFCOREZM site 683 (+) AAAG [S000265](http://www.dna.affrc.go.jp/sigscan/disp.cgi?S000265)

EBOXBNNAPA site 12 (+) CANNTG [S000144](http://www.dna.affrc.go.jp/sigscan/disp.cgi?S000144)

EBOXBNNAPA site 739 (+) CANNTG [S000144](http://www.dna.affrc.go.jp/sigscan/disp.cgi?S000144)

EBOXBNNAPA site 747 (+) CANNTG [S000144](http://www.dna.affrc.go.jp/sigscan/disp.cgi?S000144)

ERELEE4 site 653 (+) AWTTCAAA [S000037](http://www.dna.affrc.go.jp/sigscan/disp.cgi?S000037)

GATABOX site 620 (+) GATA [S000039](http://www.dna.affrc.go.jp/sigscan/disp.cgi?S000039)

GATABOX site 643 (+) GATA [S000039](http://www.dna.affrc.go.jp/sigscan/disp.cgi?S000039)

GT1CONSENSUS site 620 (+) GRWAAW [S000198](http://www.dna.affrc.go.jp/sigscan/disp.cgi?S000198)

GT1CONSENSUS site 643 (+) GRWAAW [S000198](http://www.dna.affrc.go.jp/sigscan/disp.cgi?S000198)

IBOXCORE site 620 (+) GATAA [S000199](http://www.dna.affrc.go.jp/sigscan/disp.cgi?S000199)

IBOXCORE site 643 (+) GATAA [S000199](http://www.dna.affrc.go.jp/sigscan/disp.cgi?S000199)

INRNTPSADB site 20 (+) YTCANTYY [S000395](http://www.dna.affrc.go.jp/sigscan/disp.cgi?S000395)

LECPLEACS2 site 431 (+) TAAAATAT [S000465](http://www.dna.affrc.go.jp/sigscan/disp.cgi?S000465)

MARTBOX site 395 (+) TTWTWTTWTT [S000067](http://www.dna.affrc.go.jp/sigscan/disp.cgi?S000067)

MARTBOX site 396 (+) TTWTWTTWTT [S000067](http://www.dna.affrc.go.jp/sigscan/disp.cgi?S000067)

MARTBOX site 415 (+) TTWTWTTWTT [S000067](http://www.dna.affrc.go.jp/sigscan/disp.cgi?S000067)

MYB2CONSENSUSAT site 481 (+) YAACKG [S000409](http://www.dna.affrc.go.jp/sigscan/disp.cgi?S000409)

MYBCORE site 109 (+) CNGTTR [S000176](http://www.dna.affrc.go.jp/sigscan/disp.cgi?S000176)

MYBCOREATCYCB1 site 482 (+) AACGG [S000502](http://www.dna.affrc.go.jp/sigscan/disp.cgi?S000502)

MYBST1 site 619 (+) GGATA [S000180](http://www.dna.affrc.go.jp/sigscan/disp.cgi?S000180)

MYCATRD22 site 755 (+) CACATG [S000174](http://www.dna.affrc.go.jp/sigscan/disp.cgi?S000174)

MYCCONSENSUSAT site 12 (+) CANNTG [S000407](http://www.dna.affrc.go.jp/sigscan/disp.cgi?S000407)

MYCCONSENSUSAT site 739 (+) CANNTG [S000407](http://www.dna.affrc.go.jp/sigscan/disp.cgi?S000407)

MYCCONSENSUSAT site 747 (+) CANNTG [S000407](http://www.dna.affrc.go.jp/sigscan/disp.cgi?S000407)

MYCCONSENSUSAT site 755 (+) CANNTG [S000407](http://www.dna.affrc.go.jp/sigscan/disp.cgi?S000407)

MYCCONSENSUSAT site 781 (+) CANNTG [S000407](http://www.dna.affrc.go.jp/sigscan/disp.cgi?S000407)

MYCCONSENSUSAT site 789 (+) CANNTG [S000407](http://www.dna.affrc.go.jp/sigscan/disp.cgi?S000407)

NODCON1GM site 659 (+) AAAGAT [S000461](http://www.dna.affrc.go.jp/sigscan/disp.cgi?S000461)

NODCON2GM site 249 (+) CTCTT [S000462](http://www.dna.affrc.go.jp/sigscan/disp.cgi?S000462)

NTBBF1ARROLB site 177 (+) ACTTTA [S000273](http://www.dna.affrc.go.jp/sigscan/disp.cgi?S000273)

OSE1ROOTNODULE site 659 (+) AAAGAT [S000467](http://www.dna.affrc.go.jp/sigscan/disp.cgi?S000467)

OSE2ROOTNODULE site 249 (+) CTCTT [S000468](http://www.dna.affrc.go.jp/sigscan/disp.cgi?S000468)

POLASIG1 site 213 (+) AATAAA [S000080](http://www.dna.affrc.go.jp/sigscan/disp.cgi?S000080)

POLASIG3 site 125 (+) AATAAT [S000088](http://www.dna.affrc.go.jp/sigscan/disp.cgi?S000088)

PREATPRODH site 631 (+) ACTCAT [S000450](http://www.dna.affrc.go.jp/sigscan/disp.cgi?S000450)

PROLAMINBOXOSGLUB1 site 680 (+) TGCAAAG [S000354](http://www.dna.affrc.go.jp/sigscan/disp.cgi?S000354)

PYRIMIDINEBOXOSRAMY1A site 143 (+) CCTTTT [S000259](http://www.dna.affrc.go.jp/sigscan/disp.cgi?S000259)

RAV1AAT site 448 (+) CAACA [S000314](http://www.dna.affrc.go.jp/sigscan/disp.cgi?S000314)

ROOTMOTIFTAPOX1 site 392 (+) ATATT [S000098](http://www.dna.affrc.go.jp/sigscan/disp.cgi?S000098)

ROOTMOTIFTAPOX1 site 417 (+) ATATT [S000098](http://www.dna.affrc.go.jp/sigscan/disp.cgi?S000098)

ROOTMOTIFTAPOX1 site 435 (+) ATATT [S000098](http://www.dna.affrc.go.jp/sigscan/disp.cgi?S000098)

ROOTMOTIFTAPOX1 site 453 (+) ATATT [S000098](http://www.dna.affrc.go.jp/sigscan/disp.cgi?S000098)

RYREPEATBNNAPA site 678 (+) CATGCA [S000264](http://www.dna.affrc.go.jp/sigscan/disp.cgi?S000264)

SEF4MOTIFGM7S site 207 (+) RTTTTTR [S000103](http://www.dna.affrc.go.jp/sigscan/disp.cgi?S000103)

SURECOREATSULTR11 site 734 (+) GAGAC [S000499](http://www.dna.affrc.go.jp/sigscan/disp.cgi?S000499)

**TATABOX4 site 370 (+) TATATAA** [**S000111**](http://www.dna.affrc.go.jp/sigscan/disp.cgi?S000111)

**TATABOX5 site 77 (+) TTATTT** [**S000203**](http://www.dna.affrc.go.jp/sigscan/disp.cgi?S000203)

**TATABOX5 site 205 (+) TTATTT** [**S000203**](http://www.dna.affrc.go.jp/sigscan/disp.cgi?S000203)

TATCCACHVAL21 site 601 (+) TATCCAC [S000416](http://www.dna.affrc.go.jp/sigscan/disp.cgi?S000416)

TATCCAOSAMY site 601 (+) TATCCA [S000403](http://www.dna.affrc.go.jp/sigscan/disp.cgi?S000403)

TATCCAYMOTIFOSRAMY3D site 601 (+) TATCCAY [S000256](http://www.dna.affrc.go.jp/sigscan/disp.cgi?S000256)

TGACGTVMAMY site 470 (+) TGACGT [S000377](http://www.dna.affrc.go.jp/sigscan/disp.cgi?S000377)

WBOXATNPR1 site 438 (+) TTGAC [S000390](http://www.dna.affrc.go.jp/sigscan/disp.cgi?S000390)

WBOXATNPR1 site 469 (+) TTGAC [S000390](http://www.dna.affrc.go.jp/sigscan/disp.cgi?S000390)

WBOXHVISO1 site 892 (+) TGACT [S000442](http://www.dna.affrc.go.jp/sigscan/disp.cgi?S000442)

WBOXNTCHN48 site 750 (+) CTGACY [S000508](http://www.dna.affrc.go.jp/sigscan/disp.cgi?S000508)

WBOXNTERF3 site 751 (+) TGACY [S000457](http://www.dna.affrc.go.jp/sigscan/disp.cgi?S000457)

WRKY71OS site 439 (+) TGAC [S000447](http://www.dna.affrc.go.jp/sigscan/disp.cgi?S000447)

WRKY71OS site 470 (+) TGAC [S000447](http://www.dna.affrc.go.jp/sigscan/disp.cgi?S000447)

WRKY71OS site 751 (+) TGAC [S000447](http://www.dna.affrc.go.jp/sigscan/disp.cgi?S000447)

CACT

ID CACTFTPPCA1

AC S000449

Tetranucleotide (CACT) is a key component of Mem1 (mesophyll

expression module 1) found in the cis-regulatory element in the

distal region of the phosphoenolpyruvate carboxylase (ppcA1) of

the C4 dicot F. trinervia; Y=T/C;

mesohpyll; CACT;

Flaveria trinervia

Gowik U, Burscheidt J, Akyildiz M, Schlue U, Koczor M, Streubel

M, Westhoff P. cis-Regulatory elements for mesophyll-specific gene expression in

the C4 plant Flaveria trinervia, the promoter of the C4

phosphoenolpyruvate carboxylase gene. Plant Cell. 16:1077-1090(2004)

PubMed: [15100398](http://www.ncbi.nlm.nih.gov/entrez/query.fcgi?db=PubMed&cmd=Retrieve&list_uids=15100398&dopt=Citation)

**TTATTT**

ID TATABOX5

AC S000203

"TATA box"; TATA box found in the 5'upstream region of pea (Pisum

sativum) glutamine synthetase gene; a functional TATA element by

in vivo analysis;

TATA; glutamine; synthetase;

pea (Pisum sativum);

Tjaden G, Edwards JW, Coruzzi GM

cis elements and trans-acting factors affecting regulation of a

nonphotosynthetic light-regulated gene for chloroplast glutamine

synthetase

Plant Physiol 108:1109-1117 (1995)

PubMed: [7630938](http://www.ncbi.nlm.nih.gov/entrez/query.fcgi?db=PubMed&cmd=Retrieve&list_uids=7630938&dopt=Citation); GenBank: [U22971](http://www.ncbi.nlm.nih.gov/entrez/query.fcgi?db=Nucleotide&cmd=Search&term=U22971&doptcmdl=Genbank);

**TATATAA**

ID TATABOX4

AC S000111

"TATA box"; TATA box found in the 5'upstream region of sweet

potato sporamin A gene; TATA box found in beta-phaseolin promoter

(Grace et al.); sequence and spacing of TATA box elements are

critical for accurate initiation (Grace et al.);

TATA; sporamin; phaseolin;

sweet potato (Ipomoea batatas); bean (Phaseolus vulgaris)

Grace ML, Chandrasekharan MB, Hall TC, Crowe AJ.

Sequence and spacing of TATA box elements are critical for

accurate initiation from the beta-phaseolin promoter.

J Biol Chem. 279:8102-8110 (2004).

PubMed: [14660650](http://www.ncbi.nlm.nih.gov/entrez/query.fcgi?db=PubMed&cmd=Retrieve&list_uids=14660650&dopt=Citation)

CAAT

ID CAATBOX1

AC S000028

"CAAT promoter consensus sequence" found in legA gene of pea;

CAAT; legA; seed;

pea (Pisum sativum)

Shirsat A, Wilford N, Croy R, Boulter D

Sequences responsible for the tissue specific promoter activity

of a pea legumin gene in tobacco.

Mol Gen Genet 215:326-331 (1989)

PubMed: [2710102](http://www.ncbi.nlm.nih.gov/entrez/query.fcgi?db=PubMed&cmd=Retrieve&list_uids=2710102&dopt=Citation);

TGAC

ID WRKY71OS

AC S000447

"A core of TGAC-containing W-box" of, e.g., Amy32b promoter;

Binding site of rice WRKY71, a transcriptional repressor of the

gibberellin signaling pathway; Parsley WRKY proteins bind

specifically to TGAC-containing W box elements within the

Pathogenesis-Related Class10 (PR-10) genes (Eulgem et al., 1999);

See S000390 (TTGAC), S000442 (TGACT);

WRKY; GA; MYB; W box; TGAC; PR proteins;

Oryza sativa (rice); Petroselinum crispum (parsley);

Zhang ZL, Xie Z, Zou X, Casaretto J, Ho TH, Shen QJ.

A rice WRKY gene encodes a transcriptional repressor of the

gibberellin signaling pathway in aleurone cells.

Plant Physiol. 134:1500-1513(2004)

PubMed: [15047897](http://www.ncbi.nlm.nih.gov/entrez/query.fcgi?db=PubMed&cmd=Retrieve&list_uids=15047897&dopt=Citation)

Xie Z, Zhang ZL, Zou X, Huang J, Ruas P, Thompson D, Shen QJ.

Annotations and functional analyses of the rice WRKY gene

superfamily reveal positive and negative regulators of abscisic

acid signaling in aleurone cells.

Plant Physiol. 137:176-189 (2005)

PubMed: [15618416](http://www.ncbi.nlm.nih.gov/entrez/query.fcgi?db=PubMed&cmd=Retrieve&list_uids=15618416&dopt=Citation)

Eulgem T, Rushton PJ, Schmelzer E, Hahlbrock K, Somssich IE.

Early nuclear events in plant defence signalling: rapid gene

activation by WRKY transcription factors.

EMBO J. 18:4689-4699 (1999)

PubMed: [10469648](http://www.ncbi.nlm.nih.gov/entrez/query.fcgi?db=PubMed&cmd=Retrieve&list_uids=10469648&dopt=Citation)

Eulgem T, Rushton PJ, Robatzek S, Somssich IE.

The WRKY superfamily of plant transcription factors.

Trends Plant Sci. 5:199-206. (2000)

Review.

PubMed: [10785665](http://www.ncbi.nlm.nih.gov/entrez/query.fcgi?db=PubMed&cmd=Retrieve&list_uids=10785665&dopt=Citation)

GTAC

ID CURECORECR

AC S000493

GTAC is the core of a CuRE (copper-response element) found in

Cyc6 and Cpx1 genes in Chlamydomonas; Also involved in

oxygen-response of these genes; For CuRE, see Quin and Merchant,

1995;

copper; oxygen; hypoxic;

Chlamydomonas reinhardtii

Quinn JM, Barraco P, Eriksson M, Merchant S.

Coordinate copper- and oxygen-responsive Cyc6 and Cpx1 expression

in Chlamydomonas is mediated by the same element.

J Biol Chem. 275: 6080-6089 (2000).

PubMed: [10692397](http://www.ncbi.nlm.nih.gov/entrez/query.fcgi?db=PubMed&cmd=Retrieve&list_uids=10692397&dopt=Citation)

Quinn JM, Eriksson M, Moseley JL, Merchant S.

Oxygen deficiency responsive gene expression in Chlamydomonas

reinhardtii through a copper-sensing signal transduction

pathway.

Plant Physiol. 128 :463-471 (2002).

PubMed: [11842150](http://www.ncbi.nlm.nih.gov/entrez/query.fcgi?db=PubMed&cmd=Retrieve&list_uids=11842150&dopt=Citation)

Quinn JM, Merchant S.

Two copper-responsive elements associated with the Chlamydomonas

Cyc6 gene function as targets for transcriptional activators.

Plant Cell. 7 :623-628 (1995).

PubMed: [7780310](http://www.ncbi.nlm.nih.gov/entrez/query.fcgi?db=PubMed&cmd=Retrieve&list_uids=7780310&dopt=Citation)

Kropat J, Tottey S, Birkenbihl RP, Depege N, Huijser P, Merchant

S.

A regulator of nutritional copper signaling in Chlamydomonas is

an SBP domain protein that recognizes the GTAC core of copper

response element.

Proc Natl Acad Sci U S A. 102: 18730-18735.(2005)

PubMed: [16352720](http://www.ncbi.nlm.nih.gov/entrez/query.fcgi?db=PubMed&cmd=Retrieve&list_uids=16352720&dopt=Citation)

**TATCCAC**

ID TATCCACHVAL21

AC S000416

"TATCCAC box" is a part of the conserved cis-acting response

complex (GARC) that most often contain three sequence motifs, the

TAACAAA box (see S000181), or GA-responsive element (GARE); the

pyrimidine box, CCTTTT (see S000259); and the TATCCAC box, which

are necessary for a full GA response;

gibberellin; GA; GARC;

barley (Hordeum vulgare)

Isabel-LaMoneda I, Diaz I, Martinez M, Mena M, Carbonero P.

SAD: a new DOF protein from barley that activates transcription

of a cathepsin B-like thiol protease gene in the aleurone of

germinating seeds.

Plant J. 33: 329-340 (2003)

PubMed: [12535346](http://www.ncbi.nlm.nih.gov/entrez/query.fcgi?db=PubMed&cmd=Retrieve&list_uids=12535346&dopt=Citation);

Martinez M, Rubio-Somoza I, Fuentes R, Lara P, Carbonero P, Diaz

I.

The barley cystatin gene (Icy) is regulated by DOF transcription

factors in aleurone cells upon germination.

J Exp Bot. 56: 547-556 (2005)

PubMed: [15611149](http://www.ncbi.nlm.nih.gov/entrez/query.fcgi?db=PubMed&cmd=Retrieve&list_uids=15611149&dopt=Citation)

**RESPONSE LETTER comment:**

The 626 bp promoter region of Glyma15g06080.1 that encodes miR3522 contains multiple sites for motifs:CACTFTPPCA1 (11-YACT), CURECORECR (6-GTAC), CAATBOX1 (4-CAAT), WRKY71OS (2-TGAC)and TATABOX5 (2-**TTATTT**).Two other motifs of interest with only one site each were TATABOX4(**TATATAA**)and TATCCACHVAL21 (**TATCCAC).**

YACT: cis-Regulatory elements for mesophyll-specific gene expression.

This is relevant because of miR3522 high abundance in the leaf library.

GTAC: copper- and oxygen-responsive genes

CAAT: tissue specific promoter activity of a pea legumin gene

TGAC: WRKY transcription site binding site

**TTATTT:** cis elements and trans-acting factors affecting regulation of a nonphotosynthetic light-regulated gene for chloroplast glutamine synthetase

**TATATAA:** TATA box found in beta-phaseolin promoter

**TATCCAC:** GA-responsive element
